# Genome Analysis of Two Novel *Synechococcus* Phages That Lack Common Auxiliary Metabolic Genes: Possible Reasons and Ecological Insights by Comparative Analysis of Cyanomyoviruses

**DOI:** 10.3390/v12080800

**Published:** 2020-07-25

**Authors:** Tong Jiang, Cui Guo, Min Wang, Meiwen Wang, Xinran Zhang, Yundan Liu, Yantao Liang, Yong Jiang, Hui He, Hongbing Shao, Andrew McMinn

**Affiliations:** 1 College of Marine Life Sciences, Ocean University of China, Qingdao 266003, China; jiangtongsdly@163.com (T.J.); mingwang@ouc.edu.cn (M.W.); 17865183006@163.com (M.W.); daliluyang@163.com (X.Z.); liuyundan@stu.ouc.edu.cn (Y.L.); liangyantao@ouc.edu.cn (Y.L.); yongjiang@ouc.edu.cn (Y.J.); hehui@ouc.edu.cn (H.H.); hbshao@ouc.edu.cn (H.S.); andrew.mcminn@utas.edu.au (A.M.); 2 Institute of Evolution and Marine Biodiversity, Ocean University of China, Qingdao 266003, China; 3 Key Lab of Polar Oceanography and Global Ocean Change, Ocean University of China, Qingdao 266003, China; 4 Institute for Marine and Antarctic Studies, University of Tasmania, Hobart, Tasmania 7001, Australia

**Keywords:** cyanophage, *Myoviridae*, AMGs, genome, photosynthesis

## Abstract

The abundant and widespread unicellular cyanobacteria *Synechococcus* plays an important role in contributing to global phytoplankton primary production. In the present study, two novel cyanomyoviruses, S-N03 and S-H34 that infected *Synechococcus* MW02, were isolated from the coastal waters of the Yellow Sea. S-N03 contained a 167,069-bp genome comprising double-stranded DNA with a G + C content of 50.1%, 247 potential open reading frames and 1 tRNA; S-H34 contained a 167,040-bp genome with a G + C content of 50.1%, 246 potential open reading frames and 5 tRNAs. These two cyanophages contain fewer auxiliary metabolic genes (AMGs) than other previously isolated cyanophages. S-H34 in particular, is currently the only known cyanomyovirus that does not contain any AMGs related to photosynthesis. The absence of such common AMGs in S-N03 and S-H34, their distinct evolutionary history and ecological features imply that the energy for phage production might be obtained from other sources rather than being strictly dependent on the maintenance of photochemical ATP under high light. Phylogenetic analysis showed that the two isolated cyanophages clustered together and had a close relationship with two other cyanophages of low AMG content. Comparative genomic analysis, habitats and hosts across 81 representative cyanomyovirus showed that cyanomyovirus with less AMGs content all belonged to *Synechococcus* phages isolated from eutrophic waters. The relatively small genome size and high G + C content may also relate to the lower AMG content, as suggested by the significant correlation between the number of AMGs and G + C%. Therefore, the lower content of AMG in S-N03 and S-H34 might be a result of viral evolution that was likely shaped by habitat, host, and their genomic context. The genomic content of AMGs in cyanophages may have adaptive significance and provide clues to their evolution.

## 1. Introduction

With cell numbers of up to 10^6^ cells mL^−1^ in the global oceans, unicellular cyanobacteria are amongst the most abundant photosynthetic organisms on earth and make major contributions to marine phytoplankton primary production [1,2]. *Synechococcus* and *Prochlorococcus*, the two most common marine cyanobacteria, are responsible for roughly one half of marine photosynthesis and are key players in marine biogeochemical cycles [2,3,4].

Cyanophages are viruses that infect cyanobacteria and make up an extremely abundant and genetically diverse component of marine planktonic communities. All known marine cyanophages are tailed double-stranded DNA viruses belonging to three well-defined bacteriophage families, *Myoviridae*, *Podoviridae*, and *Siphoviridae* [5]. Cyanophage infection is responsible for the mortality of a significant proportion of all cyanobacteria [6], regulating both their abundance and diversity. In coastal surface waters, more than 80% of *Synechococcus* cells were estimated to encounter infectious cyanophages on a daily basis, and ~5% to 7% of cells would become infected by viruses [6]. Viral lysis of host cells channel, or “shunt”, the photosynthetically-fixed carbon (particulate organic matter, POM) to the dissolved organic matter (DOM) pool that can be reused by cyanobacteria, driving recycling [7]. The substances released into the natural environment due to phage infection may further change the function of the environment [8,9]. 

Some cyanophages can influence marine biogeochemistry via manipulating host cell metabolism during infection. It is accomplished by the expression of auxiliary metabolic genes (AMGs) carried by phages but originating from bacterial cells. The AMGs provide supplemental support to boost host metabolic processes beneficial to phage reproduction and thereby allow an increase in energy production and a more efficient replication of the phage [10,11,12,13]. The proteins encoded by AMGs are found to participate in the host’s photosynthesis [14,15], carbon metabolism [16], nucleotide biosynthesis [10,17] and stress tolerance [18]. Cyanophage AMGs may be a special adaptive physiological mechanism that endows the phages with unique biological characteristics and a close relationship with cyanobacteria. In recent years, some AMGs have been used as marker genes for the detection of phage molecules, genetic diversity and relationships between cyanobacteria and cyanophages [19,20,21]. Among them, the photosynthesis-related genes, such as *hli* (encoding high-light-induced proteins), *psbA* (encoding the photosystem II reaction center protein D1) and *psbD* (encoding the photosystem II reaction center protein D2), are commonly found in the genome of cyanophages. Currently, all of the isolated cyanomyoviruses with complete genomes carry photosynthesis-related AMGs [11], suggesting that those genes are probably functional and essential under some conditions [22]. Besides their roles during phage infection, the genomic content of AMGs in cyanophages may also have adaptive significance and provide clues to their evolution [23].

Investigations on viral distribution in different sea areas around the world revealed high viral abundances, ranging from ~3 × 10^6^ virus ml^−1^ in the deep sea to ~10^8^ virus mL^−1^ in productive coastal waters [24]. Meanwhile, viral metagenomic studies have succeeded in discovering a large number of new viral genomes from the marine environment, far exceeding the number of existing viral genomes obtained in pure culture [25]. Compared to the large number of widespread marine viruses, there are still very few pure, cultured phages that have been isolated for research. In this study, two novel cyanophage strains belonging to *Myoviridae* (cyanomyovirus), that infected *Synechococcus,* were successfully isolated and their complete genomes were sequenced. Their genomes contain only 3–4 AMGs, far fewer than most of the cyanomyovirus genomes (>10 AMG genes) [11]. Moreover, the two new phages do not contain the highly prevalent photosynthesis-related genes (i.e., *hli*, *psbA*, and *psbD*). 

## 2. Materials and Methods

### 2.1. Cyanophage Isolation

The cyanophages S-N03 and S-H34 were isolated by liquid serial dilution from concentrated surface seawater samples collected from coastal sites (N03, 37°30.025′ N, 123°02.315′ E and H34, 32°59.98′ N, 123°59.77′ E) in the Yellow Sea. The seawater was collected and sequentially filtered by polycarbonate membrane of 3 μm (IsoporeTM 3.0 μm TSTP; Merck, Ireland) and 0.2 μm (IsoporeTM 0.2 μm GTTP; Merck, Ireland). The percolates were then filtered with a 50 kDa cartridge (Pellicon^®^ XL Cassette, Biomax^®^ 50 kDa; polyethersulfone, Millipore Corporation, Billerica, MA, USA) and concentrated through tangential flow to make the viral concentration reach 300 times the initial concentration. The viral concentrate was stored at 4 °C in the dark [26,27].

The host of the cyanophages is *Synechococcus* sp. strain MW02 (NCBI accession number KP113680). The algal culture was grown in conical flasks with f/2 seawater medium under a constant illumination of approximately 25 µmol m^−2^ s^−1^ at 25 °C in a 12-h/12-h light-dark cycle [27]. The phage enrichment was performed by adding the viral-concentrated seawater to the exponentially growing host *Synechococcus* in a ratio of 1:9. The phage-host suspension was incubated under a constant irradiance of 25 µmol m^−2^ s^−1^ at 25 °C for about 1 week until lysed host cells were observed according to the color and turbidity of the lysate. A control group was set up in parallel by replacing the viral solution with the medium [28]. The cyanophage lysates were then filtered through a 0.22 μm pore size membrane (Millex^®^-GP 0.22 μm PES; Merck, Ireland). The infection was repeated three times. The filtrate was stored at 4 °C in the dark for further tests [29]. 

### 2.2. Phage Purification

Phage purification was performed using the serial dilution method, as described previously [27]. Generally, the infectivity was tested across the serially diluted phage samples (10 times dilution over 7 orders of magnitude). The most diluted phage sample that induced host lysis was used for another round of serial dilution and infection tests. After three rounds of purification, a pure lysate with a single phage strain was theoretically produced [28]. The cyanophage was then concentrated using Amicon^®^ Ultra 15 with a 30 k-Da ultra-PL membrane (Merck, Ireland) [30]. Further purification was performed by sucrose density gradient centrifugation [27]. 

### 2.3. Host Range

The infectivity of cyanophages S-N03 and S-H34 was tested using nine *Synechococcus* strains, including *Synechococcus* WH7803, WH8102, MW02, MW03, LTWRed, LTWGreen, PSHK05, CCMP1333, PCC7002 (Table S1). The viral solution was added to each host *Synechococcus* culture in logarithmic growth phase at a volume ratio of 1:9, in triplicates. The viral solution was replaced by the medium in the control group. The mixtures were incubated under the same conditions described above. Cell lysis was monitored and compared in the control and viral solution groups every day for two weeks to examine the infectivity. 

### 2.4. Morphological Study by Transmission Electron Microscopy

The 20 μL purified phage suspensions were placed onto a 200-mesh copper grid and stained by adding a drop of 1% (*w*/*v*) phosphotungstic acid (pH 7.2) for 10 min [29]. The grids were examined using a transmission electron microscope (JEOLJEM-1200EX, Japan) at 100 kV to reveal the cyanophage structural characteristics and dimensions [31].

### 2.5. Genome Sequencing and Assembly

Phage DNA was extracted from the sucrose density gradient-purified phages using a TIANamp Virus DNA Kit (TIANGEN) [27]. A total of 1 µg DNA per sample was used as input for the DNA sample preparations. Sequencing libraries were generated using NEBNext^®^ Ultra™ DNA Library Prep Kit for Illumina (NEB, USA). The whole genomes of S-N03 and S-H34 were sequenced using Illumina NovaSeq PE150 by an ABI 3730 automated DNA sequencer. The reads containing >40% low-quality bases (mass value ≤20), >10% N content, overlap with the adapter for >15 bp with less than 3 mismatches, were removed. The reads were assembled with SOAP denovo [32], SPAdes [33], and Abyss [34] software packages. The assemblies from the three software packages were then integrated with CISA software to select the one with the least scaffolds. Gapcloser and GapFiller were used to fill the assembly gaps [27,35].

### 2.6. Genome Annotation and Analysis 

The open reading frames (ORFs) in the genomes of cyanophage S-N03 and S-H34 were predicted using GeneMarkS [36], GLIMMER [37] and RAST (Rapid Annotation using Subsystem Technology) [38]. The predicted ORFs were translated into amino acid sequences and their homologous genes were searched in the NCBI (National Center for Biotechnology Information) non-redundant protein database by BLASTp [39,40]. The protein domains were predicted and analyzed by InterPro [41] and CDD [42]. tRNA scan-SE was used to identify transfer RNA (tRNA) genes [43], and RNAmmer was used to predict ribosomal RNA in the full genome sequence [44]. Genome mapping was performed using DNAplotter (version 17.0.1). 

An AMG database was created, which summarized protein sequences from 33 AMGs that were chosen based on prior recognition and extracted from various cyanomyovirus genomes [11,45]. A total of 337 genomes were downloaded from the NCBI database, which include all isolated cyanomyovirus with complete genomes available at the time of analysis (Table S2). Only one representative genome, when there were phages of the same name with the average nucleotide identity (ANI) greater than 95% was kept. Finally, 81 representative genomes of cyanophages were selected. Gene identity was assigned to a corresponding AMG gene when the BLASTp E-value ≤10^−5^, sequence identity ≥35%, and the query cover ≥60% [11]. The genome sequences of S-N03 and S-H34 were deposited in the GenBank database under accession number MT162466 and MT162467, respectively.

### 2.7. Comparative Analysis of Cyanophage Genomes

The ViPTree server was used to generate a proteomic tree based on the genome-wide sequence similarities computed by tBLASTx [46,47]. All related viruses contained in the Virus-Host Database were used to establish a circular tree [48]. The 37 closest phages in the circular tree were then selected to establish a rectangular tree with phage S-N03 and S-H34 for subsequent comparison and analysis. The genome sequences of S-N03 and S-H34 were compared with that of phage S-B68 by tBLASTx using ViPTree. Meanwhile, phylogenetic analysis with other related phages were carried out using the amino acid sequences of DNA polymerase and terminase large subunit by the ClustalW program. The maximum-likelihood (ML) phylogenetic tree was constructed by genetic analysis software MEGA (Version 7.0.18) [49,50]. The bootstrap values were based on 1000 replicates. The average nucleotide identity (ANI) was calculated using OrthoANI (Average Nucleotide Identity by Orthology) [51] and JSpeciesWS Online Service [52].

## 3. Results and Discussion

### 3.1. Host Range and Phage Morphology

The host of cyanophage S-N03 and S-H34 is PE-type (phycoerythrin-only) *Synechococcus* sp. strain MW02, which belongs to subcluster 5.1 clade IX and was originally isolated from Hong Kong estuarine waters [53]. The cross-infectivity test showed that both S-N03 and S-H34 infected the other three PE-type *Synechococcuses* belonging to subcluster 5.1 clade II, V and subcluster 5.2 (Table S1). The transmission electron microscopy examination showed that S-N03 and S-H34 displayed icosahedral heads of 97 and 88 nm in diameter and contractile tails of 138 and 129 nm in length, respectively. Their sizes are within the range of the previously isolated cyanomyoviruses (Figure S1).

### 3.2. General Genomic Features

Cyanophage S-N03 and S-H34 both contain a circular double-stranded DNA genome revealed by the terminal analysis that showed no protruding cohesive. The genome sizes of S-N03 and S-H34 are 167,069-bp and 167,040-bp, which are the sixth and seventh smallest genomes among the 81 representative cyanomyoviruses (Table 1). The G + C content of the genomes S-N03 and S-H34 are both 50.1%, two of the only four published cyanomyoviruses genomes (S-B68, S-CBWM1, S-N03 and S-H34) with G + C contents close to 50%. The G + C content of *Prochlorococcus* phages (34.3–40.7%) is generally lower than that of *Synechococcus* phages (35.4–51.7%). In the 81 representative cyanomyoviruses, about 71.4% of *Prochlorococcus* phages have G + C contents of less than 38.1%, while most of the *Synechococcus* phages have G + C contents between 38% and 45% and only 11.1% have G + C contents of less than 38.1% (Table 1). Studies have shown that the genomes of some organisms that depend on the survival of the host, such as bacteria, phages, and plasmids, are often rich in A + T, that is, the G + C content is low. This may be due to the differential cost of related metabolites in the cell and the limited availability of G and C relative to A and T/U [54]. Neutral bias can also explain the higher A + T content of phages, because the depletion of host bacterial resources may result in the systematic insertion of more abundant A and T nucleotides [54]. Moreover, the G + C content of the phage may be also affected by that of their host. A positive correlation has been observed between G + C content of bacteriophage and their host [55]. In this study, the G + C contents of *Synechococcus* marinus WH8102 and WH7803 that can be infected by S-H34 and S-N03 were 59.2% and 60.2%, respectively (the whole genome of their host MW02 was not published), which was at a relatively high level in the G + C content range of marine *Synechococcus* (~50–60% G + C content) [56]. Such high G + C content of a host might be associated with the high G + C content of the cyanophages, although more evidence is needed to prove this point. Thus, we inferred that the higher G + C content implied that S-N03 and S-H34 may have experienced independent evolutionary routes compared to the cyanophages of lower G + C values and evolved specific genomic traits that adapted to their hosts and their surrounding environments.

In order to understand whether there is a relationship between the genome size and G + C content in the viral genome, the Spearman correlation analysis was performed based on the 81 representative genomes of cyanophages. The relationship between genome size and G + C content has been studied for bacteria but seldomly investigated on viruses [57]. Interestingly, we found a significant negative correlation between genome size and G + C content of cyanomyoviruses (r = −0.34, *p* < 0.01, Table 2). This is in contrast to bacteria and archaea, which were shown to have positive correlations between G + C values and genome size, but consistent with the result obtained in bacteriophages [55,57]. Such a negative relationship in cyanomyoviruses is still thought to be related to their adaptive evolution. If a phage genome is large and enriched with G + C at the same time, higher energy cost and limited availability of G/C could constrain phage-DNA replication, which does not comply with the life strategy of viruses. 

**Figure 1:**
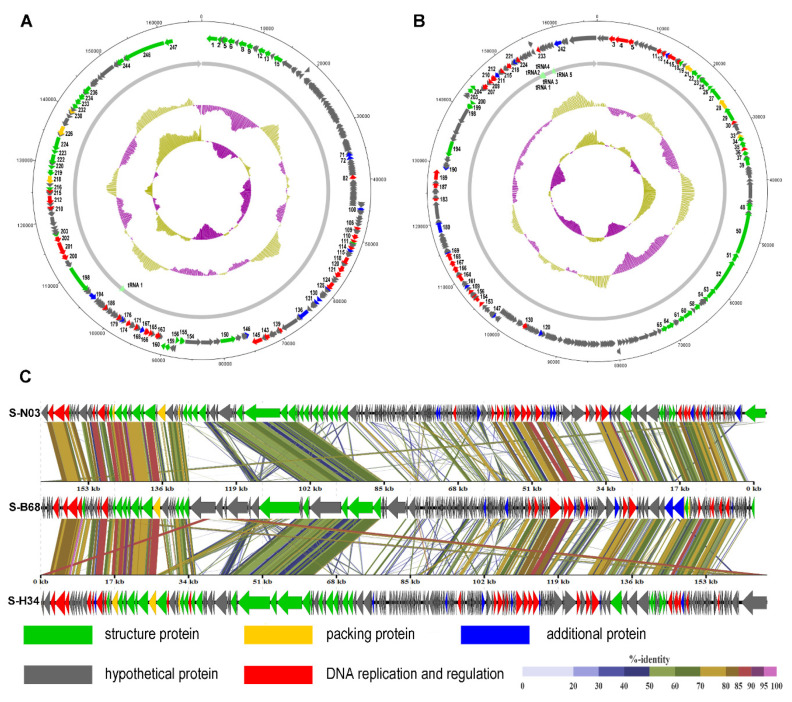
Genome map and functional annotation of the predicted proteins of cyanophage (**A**) S-N03 and (**B**) S-H34. The number next to the arrow indicates the ORF number. (**C**) Genome-wide comparison of phages S-N03, S-H34 and S-B68.

A total of 247 and 246 potential ORFs were identified in the S-N03 and S-H34 genomes, respectively (Table S3 and S4). Functional annotation of predicted ORFs in the NCBI non-redundant protein database showed that only 72 (29.15%) were assigned to specific functions in S-N03 (*E*-value < 10^−5^), while the rest 175 (70.85%) were predicted to encode hypothetical proteins, due to incomplete genomic information of the cyanophage in database [69]. Similarly, 70 (28.46%) predicted ORFs were assigned to specific functions in S-H34, while the rest 176 (71.54%) were predicted to encode hypothetical proteins. All predicted ORFs can be divided into five functional groups, including structuring (S-N03, 31ORFs and S-H34, 28 ORFs), packaging (S-N03, 3ORFs and S-H34, 3 ORFs), DNA replication and regulation (S-N03, 26 ORFs and S-H34, 29ORFs), hypothetical protein and additional functions related to physiological activity (12 ORFs in S-N03 and 10 ORFs in S-H34) (Figure 1A,B). 

The functional annotation of phage structural proteins is highly dependent on the sequence similarity to proteins of other phages that were detected in respective viral particles [70,71]. The putative structural proteins in S-N03 and S-H34 were the baseplate, the tail tube, the tail sheath, the tail fibers, the tail completion proteins and neck proteins. The packaging modules of both S-N03 and S-H34 contain three ORFs, including terminase large subunit, terminase small subunit and major capsid protein. The DNA replication and conditioning module contained a wide variety of categories, including DNA primase, RNA polymerase, single-stranded DNA binding protein UvsY, endonuclease, DNA polymerase, exonuclease, ribonuclease H, ribonucleoside-diphosphate reductase alpha subunit (NrdA), and ribonucleotide diphosphate reductase beta subunit (NrdB). Among these, NrdA (S-N03: ORF202, S-H34: ORF4) and NrdB (S-N03: ORF201, S-H34: ORF3) are involved in DNA synthesis by converting nucleotides into deoxynucleotides, and can be found in all organisms [72,73]. In a marine environment with limited phosphorus content, obtaining sufficient free nucleotides is critical for DNA synthesis [72,74,75]. With ribonucleotide reductase (NrdA, NrdB) and thymidylate synthase (S-N03:ORF171, S-H34:ORF215), the rate of DNA synthesis of T4-like phage could be increased 10-fold compared to a system without these enzymes [76].

Additional proteins modules are mainly related to metabolism and regulation. In addition to AMGs (detailed results and discussions are shown in Section 3.5), S-N03 and S-H34 also have regulatory genes, such as genes encoding serine/threonine kinase (PSKs) PknB (S-N03: ORF136, S-H34: ORF180), serine/threonine phosphatase (S-N03: ORF100, S-H34: ORF147) and endolysins. PknB is a typical Ser/Thr kinase, which catalyzes the transfer of the gamma-phosphoryl group on the ATP molecule to the Ser/Thr residue of the protein substrate. It is involved in regulating many biological processes, including purine and pyrimidine biosynthesis, cell wall metabolism, antibiotic resistance, peptidoglycan synthesis, cell division, transcription, stress response and metabolic regulation [77,78,79]. Ser/Thr phosphatase (PSPs) are responsible for dephosphorylation of phosphoprotein substrates, which is the reverse process of Ser/Thr kinase catalysis. They participate in many cell pathways that regulate cell reproduction and programmed death [80]. The reversible phosphorylation of proteins is accomplished by opposing activities of kinases and phosphatases [80]. S-N03 and S-H34 also contain endolysin with amino acid identities of 51.75% (97% coverage) with that of cyanophage S-B68. Endolysins are enzymes produced by phages. They are responsible for catalyzing the hydrolysis of the peptidoglycan in the bacterial cell wall and rupturing the cell at the end of the virulence cycle [81].

### 3.3. tRNA Genes

Apart from host-like genes, cyanomyoviruses have also incorporated tRNA genes into their genomes. In this study, only one tRNA gene (Ans) was identified in the genome of S-N03 and five tRNA genes (Tyr, Asp, Val, 2× Ans) were identified in the genome of S-H34 (Table 3). The number and types of tRNA genes is variable in different Cyanomyoviruses (Table 1), which is a result of phage-host co-evolution, driven by the optimal codon usage [82]. The tRNAs carried in phage genomes match codons highly used by the phage and poorly used by the bacterial host during the infection [83]. They may augment the expression of late phage genes encoding structural proteins, such as phage capsid and tail proteins [56,84,85]. Therefore, the tRNA genes carried by S-N03 and S-H34 may contribute to phage protein synthesis and help the phage to adapt to a particular host or environment. Among the 81 published cyanomyoviruses, the number of tRNA contained in *Prochlorococcus* phages (0–4 tRNA) is significantly less than that in *Synechococcus* phages (1–36 tRNA); S-N03 is one of the *Synechococcus* phages that contains the least amount of tRNA. It has been proposed that the number of tRNAs genes is closely associated with differences of G + C content between phage and host: more tRNAs may increase the translation efficiency when infecting a host with higher G + C content, and potentially expand their potential host range while maintaining relatively lower G + C content in their genomes [56]. As such, the tradeoff between the G + C content and the occurrence of tRNA genes may result in the relatively low number of tRNAs and the wide host range of S-N03 and S-H34, given their relatively high G + C content (50.1%; Table 2) which is close to that of their hosts (marine *Synechococcus* of ∼50–60% G + C) [56]. 

### 3.4. Genome Comparison and Phylogenetic Analysis

**Figure 2:**
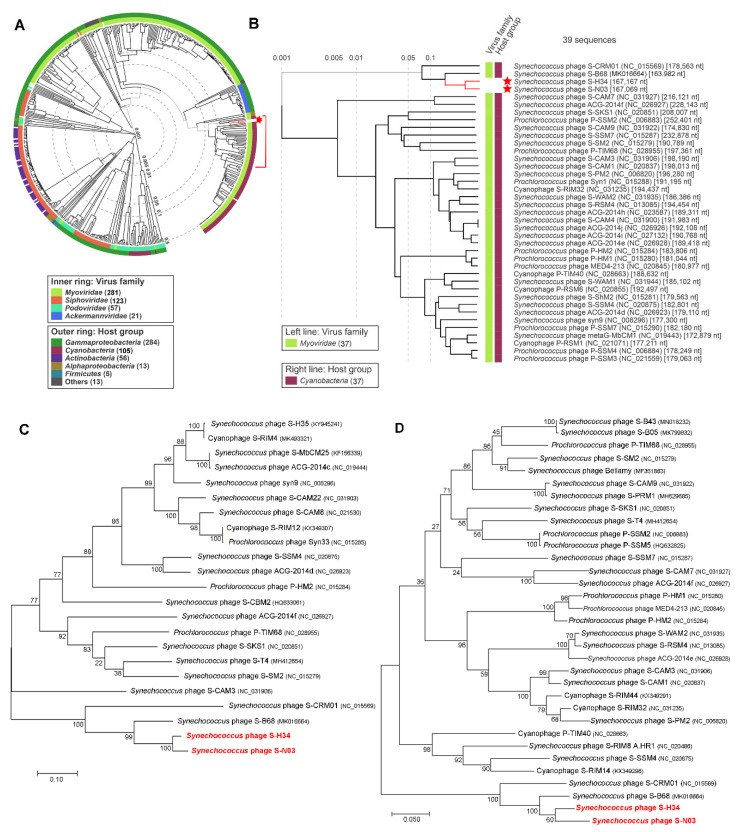
Phylogenetic analysis. (**A**,**B**) Phylogenetic analysis with other related phages using the genome-wide sequence similarities computed by tBLASTx. These evolutionary trees have no roots. (**C**,**D**) Phylogenetic ML tree with other related phages based on the amino acid sequences of (**C**) DNA polymerase and (**D**) terminase large subunit. The trees were constructed in MEGA version 7 by the ML method with 1000 bootstrap replicates. The percentage of replicate trees in which the associated taxa clustered together in the bootstrap test are shown next to the branches. The scale bar represents 0.1 (**C**) or 0.05 (**D**) amino acid substitution per site.

Comparative genome analysis was undertaken to reveal the divergence of the nucleotide sequence of S-N03 and S-H34 from other cyanophages. The “proteomic tree”, based on the genome-wide similarities, showed that the phages with the closest phylogenetic relationship to S-N03 and S-H34 all belong to cyanomyoviruses (Figure 2A,B). S-N03 and S-H34 have the closest genetic relationship with each other and group into the branch that also contains S-B68 and S-CRM01 and are distant from other cyanophages. The phylogenetic analyses of S-N03, S-H34 and other selected dsDNA viruses, based on the DNA polymerase and terminase large subunit sequences using the maximum likelihood method (ML), showed similar branching positions of S-N03 and S-H34 (Figure 2C,D). They share a highest similarity to each other with a nucleotide identity of 84.21% (coverage rate 67.81%). In this cluster, a lower similarity of S-N03 and S-H34 was obtained to S-B68 (identity of 70.54% and 72.18%, respectively) and S-CRM01 (identity of 63.71% and 64.76%, respectively), indicating that S-N03 and S-H34 are novel cyanophages. 

Among these four isolates of cyanophage, S-H34, S-N03 and S-B68 are all marine lytic phages and have higher similarities, while S-CRM01 is a freshwater strain. Therefore, the genomes of S-N03 and S-H34 were compared with S-B68 (Figure 1C). The three genomes were found to share a high similarity in some proteins coded by the conserved genes, including DNA polymerase, terminase large subunit, and the major capsid protein, with 75.31–93.65% identity on amino acid level (Figure 1C). The differences among the genomes come mainly from a number of hypothetical proteins. Moreover, S-B68 is distinguished from S-H34 and S-N03 by the metabolic genes it encodes. It could be speculated that these differences arise due to the difference in host species because S-B68 have a different host from S-H34 and S -N03 [86].

### 3.5. Auxiliary Metabolic Genes (AMGs)

**Figure 3:**
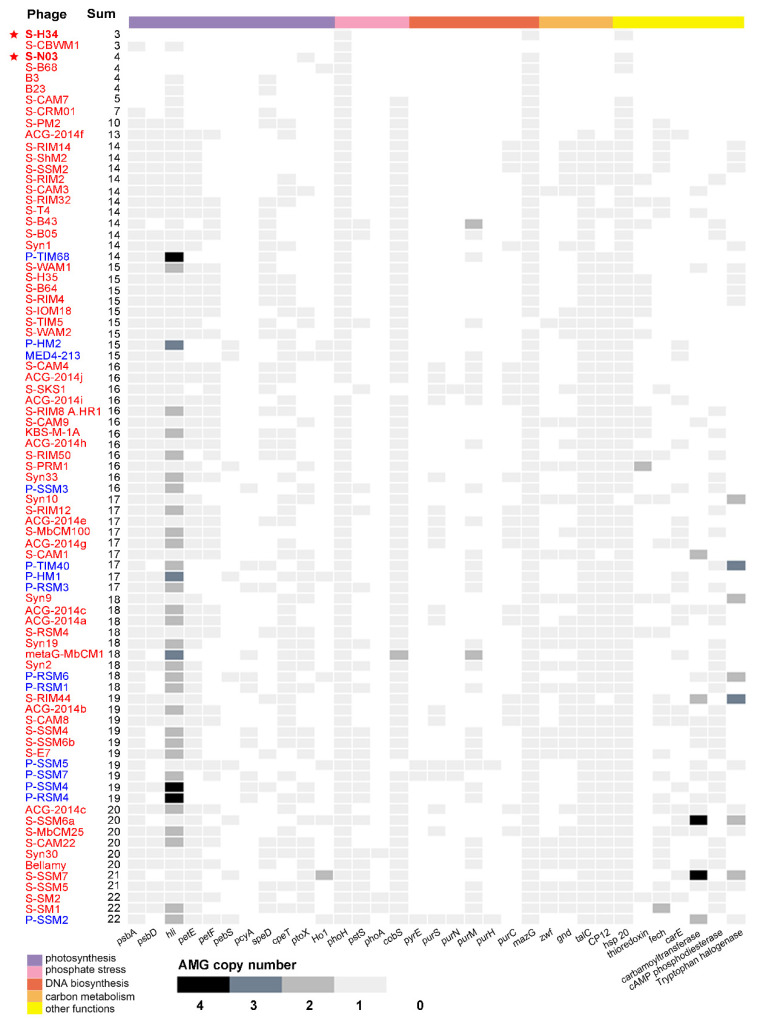
A heat map of gene copy number matrix for 33 auxiliary metabolic genes (AMGs) across 81 representative genomes of isolated cyanomyoviruses with available complete genome in NCBI. The AMG content was listed in an ascending order. The names of the cyanophages are colored separately by the originally isolated genus of the host: *Synechococcus* is red and *Prochlorococcus* is blue.

AMGs are commonly found in the genome of cyanophages. Among 81 cyanomyoviruses with available complete genomes, 92.6% are found to contain more than 5 AMGs (Figure 3). However, the newly isolated cyanophage S-N03 contains only 4 AMGs (*hsp*, *MazG*, *ptoX*, *phoH*) and S-H34 contains only 3 AMGs (*hsp*, *MazG*, *phoH*). The latter one is the phage with the least number of AMGs genes isolated so far (Figure 3). All of the AMGs found in S-N03 and S-H34 genomes are highly conserved genes among cyanophages.

#### 3.5.1. *MazG* Gene (Pyrophosphatase)

MazG protein, the pyrophosphatase, is known as a regulator of nutrient stress and programmed cell death in *E. coli* [20]. The phage-encoded MazG was proposed to regulate the cellular level of ppGpp and, therefore, to affect transcription and translation in the host and extend the period of cell survival under the stress of phage infection [59,87]. However, a recent study showed that the purified cyanophage S-PM2 MazG has no binding or hydrolysis activity to (p)ppGpp [88]. Instead, dGTP and dCTP seem to be the preferred substrates for this protein, and affinity of the viral MazG for dGTP and dCTP is higher than their host counterparts. This may partially explain the lower G + C content of cyanophage genomes (37.7%) than that of the *Synechococcus* host genomes (60.2%), and it is consistent with preferential hydrolysis of deoxyribonucleotides in the host *Synechococcus* genome of high G + C content [88]. However, whether such a mechanism is applicable to cyanophages whose genomes generally have a similar G + C content with their hosts, such as S-H34 and S-N03 in this study, has yet to be determined. *MazG* is a highly conserved gene in cyanopodoviruses and cyanomyoviruses that infect *Synechococcus*. Only S-TIM5 and S-CBWM1 lack *MazG* in the 81 cyanomyoviruses examined (Figure 3). Previous research used the pyrophosphate nucleotide hydrolase gene *MazG* to prove that cyanophages are globally distributed. Despite the widespread presence of *MazG* gene in cyanophages, they have a small effective population size, indicative of rapid lateral gene transfer [20]. The phylogenetic trees based on *MazG* gene from previous studies showed that *Prochlorococcus* and *Synechococcus* phage *MazG* genes do not cluster with their hosts’ *MazG*, suggesting that this gene may be not obtained from the host but acquired by lateral gene transfer from other sources [20,88].

#### 3.5.2. *phoH* Gene (P-Starvation Inducible Protein)

**Figure 4:**
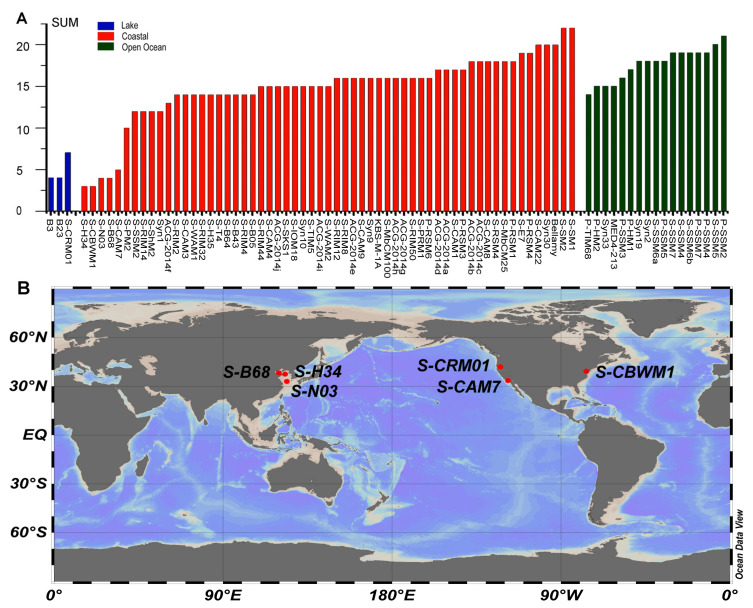
(**A**) A histogram showing the distribution of AMG content in 81 cyanomyoviruses from different type of habitat (coastal areas, open oceans and lakes). (**B**) The isolation sites of the published cyanophages with AMGs content less than 10.

As the most prevalent phosphate-regulating gene in the genomes of cyanophages, *phoH* is present in 80 of 81 related cyanomyoviruses (except S-SKS1, Figure 4). Although its function is still unclear, it has been used as a molecular marker for describing viral diversity due to its universality [21,89]. In this study, the host of cyanophage S-N03 and S-H34, *Synechococcus* sp. strain MW02, was isolated from a Hong Kong estuarine site (affected by the Pearl River flows) where phosphorus limitation is usually present [53,90]. As such, these genes may play a role in regulating the phosphate uptake of the hosts from the environment.

#### 3.5.3. *Hsp* Gene (Heat Shock Protein)

Heat shock proteins (Hsps) are clusters of proteins that are induced in response to physical and chemical environmental stresses. They can facilitate cellular recovery from the damage caused by participating in protein translocation, re-folding and degradation, and are known as “molecular chaperones” [91]. Most of the heat shock proteins found in bacteriophages are small (sHsps), which can suppress protein aggregation and protect against cell stress, and are generally active as large oligomers consisting of multiple subunits [92,93]. Specifically, the heat shock protein family in phages might be important for scaffolding during maturation of the capsid [45]. Only 4 of the 81 cyanophages (S-CBWM1, B2, B23 and Syn10) did not contain the *Hsp* gene (Figure 3). Previous study has shown that cyanophage’s sHSPs form a monophyletic clade phylogenetically closer to bacteria than to cyanobacteria, while the host cyanobacterial sHSPs sequences forms a monophyletic clade closer to plants [93]. Such phylogenetic relationships point to horizontal gene transfer events that probably occurred millions of years ago. This means that the cyanophage *sHsp* gene has evolved independently and differently from its actual host cyanobacteria, but it still co-evolved with the host cyanobacteria in the pseudo- or lysogenic stage [93]. 

#### 3.5.4. *ptoX* Gene (Plastoquinol Terminal Oxidase)

PTOX is an enzyme that mediates the electron flow from plastoquinol to oxygen. It exerts a variety of effects on the development and functioning of plant chloroplasts, including carotenoid biosynthesis, photoprotection and chlororespiration [60,94]. It does not exist in all photosynthetic organisms, but it is widely distributed among different strains of cyanobacteria [95]. The *ptoX* genes are also widespread among marine cyanomyoviruses (Figure 3). By carrying the *ptoX* gene, the cyanophage may have another way of preventing photodamage other than the *psbA* route [60]. PTOX can oxidize the plastoquinol produced by chloroplast NAD(P)H quinone oxidoreductase, which called chlororespiration. In the phage genome, PTOX is often arranged adjacent to NAD(P)H quinone oxidoreductase [95]. And in the genome of S-N03, NAD(P)H oxidoreductase appears upstream of PTOX (Figure 1A, Table S3). This indicates that NAD(P)H oxidoreductase and PTOX represent functional units in these cyanobacteria and may be transcription units. The phylogeny of the PTOX protein of the host and the cyanophage implies that although both *Synechococcus* and *Prochlorococcus* hosts and the cyanophage *ptoX* gene may share a common ancestor, they have evolved independently since then [60]. 

#### 3.5.5. Lack of Photosynthetic AMGs

The prevalence of AMGs in the 81 sequenced cyanomyoviruses of *Synechococcus* and *Prochlorococcus* (including the 2 strains in this study) is shown in Figure 3. It shows that all the sequenced cyanomyoviruses carry at least one photosynthesis-related AMG. Some of the genes, such as the *psbA*, *psbD*, and *hli*, are common in the genomes of cyanomyoviruses. For example, it was found that 99% of the phage genomes contain *hli*, 95% contain *psbA*, and 76% contain both *psbA* and *psbD*. However, unlike all other known cyanomyoviruses, the genome of the novel strain S-H34 did not contain photosynthesis-related AMGs. The novel strain S-N03 only contains one photosynthesis-related AMG *ptoX* (plastoquinol terminal oxidase), but lacks the common *psbA*, *psbD*, and *hli*. 

The D1 and D2 proteins encoded by the *psbA* and *psbD* genes are core reaction-center proteins in photosystem II and participate in photochemical reactions. The D1 protein produced by the host turns over rapidly under high light and declines during phage infection [12,86,96]). Therefore, the expression of phage *psbA* gene during infection, as confirmed in previous studies, can bolster the host’s photosynthesis [12]. The high light-induced protein encoded by *hli* serves an important role in preventing cellular light damage by redirecting excessive light energy and protecting the photosynthetic apparatus [97]. By supplementing host photosynthesis, the phage photosynthetic AMGs ensures the energy required for phage maximum production and thus enhances their fitness [14]. It has been suggested that some phage photosynthetic AMGs (i.e., *psbA* and *hli*) have become an integral part of the phage genome as they are co-transcribed with the essential, highly expressed phage capsid genes surrounding the photosynthesis genes [12]. As such, the absence of the common photosynthetic AMGs in S-N03 and S-H34 infers their distinct evolutionary route. It also suggests that the energy for morphogenesis during phage production might be obtained from sources other than those strictly dependent on the maintenance of photochemical ATP under high light. 

It has been suggested that the number of photosynthesis AMGs (i.e., *psbA* and *hli*) and the optimal combination required by the phage may be determined by the light level [92]. The cyanophage fitness enhancement conferred by the phage photosynthesis genes only occurrs under a certain range of high light [86,98]. For example, a novel agent-based model shows that the phage photosynthesis genes are not necessary at a depth of 30 m, and the optimal photosynthesis gene combination in the phage was simplified to 0 *psbA* and 1 *hli* at a depth of 120 m [98]. In addition, the length of the latent period of infection has also been speculated to determine the presence or absence of *psbA* in a phage genome [99]. Therefore, the distinct genomic feature of S-H34, with an absence of all photosynthetic AMGs, might be the result of environmental adaptation and/or their own physiological characteristics.

#### 3.5.6. Low AMG Contents in S-H34 and S-N03

Of the known cyanomyoviruses, less than 10% carry ≤10 AMGs in their genomes (Figure 3). S-H34 is one of the strains with by far the fewest AMGs (3 AMGs, equal to that of S-CBWM1). Intriguingly, from the phylogenetic analysis, it was noticed that S-N03 and S-H34 have the closest relationships with S-B68 and S-CRM01 (Figure 2B), which also have fewer AMGs (4 AMGs in S-B68 and 7 AMGs S-CRM01). This suggests that AMG content may be related to the genetic relationship. However, large variations of AMG content in phylogenetically closely-related cyanomyovirus genomes have also been demonstrated in previous studies [11,27]. It has been suggested that both vertical and horizontal evolution determine the AMGs content: the highly conserved AMGs across cyanomyoviruses are likely maintained by vertical inheritance while those occasional AMGs may be due to horizontal evolution under different selection pressures such as environmental condition and host type. 

In order to investigate the distribution of AMG content in different environments, the cyanophages were divided according to their location type (coastal, open ocean and lake) and plotted with their corresponding number of AMGs (Figure 4A). Although no significant difference in AMG number was identified between coastal and the open ocean regions, it is clear that the cyanophages with low AMG content were all isolated from relatively eutrophic areas such as coastal regions and lakes (Figure 4A). Further analysis using the six published cyanomyoviruses with AMG content less than 10 showed that these phages were all isolated from mid-latitude regions of 30–40°N (Figure 4B), and their hosts were all *Synechococcus* (Table 4). Compared with cyanomyoviruses isolated from *Prochlorococcus*, genomes of *Synechococcus* had a lower AMG content (Figure 3, Table 1). Data presented here are consistent with previous studies that also proposed an association of AMG content with location and the host genus [11]. Moreover, neither S-N03 nor S-H34 showed strict host specificity and the ability to infect other *Synechococcus* strains besides their host *Synechococcus* MW02, which coincided with the speculation that the expansion of the host range may be also accompanied by a decrease in AMG content in some cases [11]. 

The low number of AMGs in the two phages may also be related to the genomic features of small genome size and high G + C content. By performing the correlation analyses on the number of AMGs, genome size and G + C values of the 81 representative cyanomyoviruses, a significantly negative correlation was obtained between the number of AMGs and GC% (*r* = −0.272, *p* < 0.05, Table 2) However, although the AMG content did not correlate with the genome size in our dataset, the newly acquired genes fixed in the viral genome is usually at the cost of larger genome size [10]. Collectively, the lower content of AMG in S-N03 and S-H34 might be a result of viral evolution that was likely shaped by the habitat (eutrophic seawater), host type and range (*Synechococcus* phages with relatively wide host range), and genomic features (small genome size and high G + C content). However, more evidence is still needed to elucidate the regulation of AMG type and content in cyanophages.

## 4. Conclusions

In this study, two novel *Synechcoccus* phages, S-N03 and S-H34, were isolated, and their complete genomes were sequenced and analyzed. Both phages have relatively small genomes with high G + C content. Fewer AMGs than other cyanomyoviruses and an absence of common photosynthesis-related genes were also observed, which imply their different evolutionary routes were shaped by habitat types and host preference, and give clues to their likely ecological functions. Due to the limited information on genes and proteins in the cyanobacterial gene database, isolation and sequencing of more cyanophages from different environments are urgently needed. The cyanophage genomic information can contribute to further research on the interaction between cyanophage and their hosts in aquatic environments, and provide insights into viral adaptive evolution and ecological functions. 

## Figures and Tables

**Table 1:** General genomic features of 81 representative cyanomyoviruses.

Host	Phage	Genome Size (bp)	GC (%)	AMG	tRNA	NCBI Taxonomy ID	No. Isolates	Accession	RefSeq Accession	Ref.	Submission Date
*Synechococcus*	S-PM2	196,280	37.8	10	25	238854	2	AJ630128.1	NC_006820.1	[58]	2004
	Syn9	177,300	40.6	18	6	382359	1	DQ149023.2	NC_008296.2	[59]	2005
	S-RSM4	194,454	41.1	18	12	555387	1	FM207411.1	NC_013085.1	[60]	2008
	Syn1	191,195	40.6	14	6	444861	1	GU071105.1	NC_015288.1	[45]	2009
	Syn19	175,230	41.3	18	6	445684	1	GU071106.1	NC_015286.1	[45]	2009
	Syn33	174,285	39.6	16	5	444878	1	GU071108.1	NC_015285.1	[45]	2009
	S-SM1	174,079	41.1	22	6	444859	1	GU071094.1	NC_015282.1	[45]	2009
	S-SM2	190,789	40.4	22	11	444860	1	GU071095.1	NC_015279.1	[45]	2009
	S-ShM2	179,563	41.1	14	1	445683	1	GU071096.1	NC_015281.1	[45]	2009
	S-SSM5	176,184	40	21	4	445685	1	GU071097.1	NC_015289.1	[45]	2009
	S-SSM7	232,878	39.1	21	5	445686	1	GU071098.1	NC_015287.1	[45]	2009
	Syn2	175,596	41.3	18	6	536473	1	HQ634190.1	-	-	2010
	Syn10	177,103	40.6	17	6	536472	1	HQ634191.1	-	-	2010
	Syn30	178,807	39.9	20	6	536474	1	HQ634189.1	NC_021072.1	-	2010
	S-SSM2	179,980	41.1	14	1	536464	1	JF974292.1	-	-	2010
	S-SSM4	182,801	39.4	19	3	536466	1	HQ316583.1	NC_020875.1	-	2010
	S-SSM6a	232,883	39.1	20	5	682650	1	HQ317391.1	-	-	2010
	S-SSM6b	182,368	39.4	19	3	682651	1	HQ316603.1	-	-	2010
	S-CAM1	198,013	43	17	8	754037	6	HQ634177.1	NC_020837.1	-	2010
	S-CAM8	171,407	39.3	19	5	754038	2	HQ634178.1	NC_021530.1	-	2010
	S-CRM01	178,563	39.7	7	34	1026955	1	HQ615693.1	NC_015569.1	[61]	2010
	S-RIM8	171,211	40.6	16	8	869724	13	JF974288.1	NC_020486.1	[62]	2010
	S-SKS1	208,007	36	16	12	754042	1	HQ633071.1	NC_020851.1	-	2010
	KBS-M-1A	171,744	40.6	16	8	889950	1	JF974293.1	-	-	2010
	S-IOM18	171,797	40.6	15	7	754039	1	HQ317383.1	NC_021536.1	-	2010
	metaG-MbCM1	172,879	39.8	18	2	1079999	1	JN371769.1	NC_019443.1	-	2010
	S-TIM5	161,440	40.5	15	10	1137745	1	JQ245707.1	NC_019516.1	[63]	2011
	ACG-2014c	176,043	39.1	20	4	1079998	5	JN371768.1	NC_019444.1	[64]	2011
	ACG-2014a	171,282	39.4	18	5	1493507	24	KJ019026.1	-	[65]	2013
	ACG-2014b	172,688	39.1	19	5	1493508	18	KJ019134.1	NC_027130.1	[65]	2013
	ACG-2014d	179,110	40.3	18	3	1493509	45	KJ019136.1	NC_026923.1	[65]	2013
	ACG-2014e	189,418	38.9	17	8	1493510	3	KJ019156.1	NC_026928.1	[65]	2013
	ACG-2014f	228,143	41.6	13	2	1493511	41	KJ019059.1	NC_026927.1	[65]	2013
	ACG-2014g	174,885	39.3	17	5	1493512	1	KJ019071.1	NC_026924.1	-	2013
	ACG-2014h	189,311	40.5	16	7	1340810	1	KF156338.1	NC_023587.1	[64]	2013
	ACG-2014i	190,768	39	16	8	1493513	1	KJ019082.1	NC_027132.1	[65]	2013
	ACG-2014j	192,108	38.6	16	7	1493514	2	KJ019069.1	NC_026926.1	[65]	2013
	S-MbCM25	176,044	39.1	20	4	1340811	1	KF156339.1	-	[64]	2013
	S-MbCM100	170,438	39.4	17	5	1340812	1	KF156340.1	NC_023584.1	[64]	2013
	S-RIM2	175,430	42.2	14	6	869662	62	HQ317292.1	NC_020859.1	[66]	2016
	S-RIM12	173,821	39.6	17	5	1278402	21	KX349307.1	-	[66]	2016
	S-RIM14	179,558	41.1	14	1	1278423	9	KX349298.1	-	[66]	2016
	S-RIM32	194,437	39.9	14	11	1278479	1	KU594606.1	NC_031235.1	[11]	2016
	S-RIM44	197,629	40.3	19	5	1278485	8	KX349291.1	-	[66]	2016
	S-RIM50	174,307	40.3	16	8	687803	1	KU594605.1	NC_031242.1	[11]	2016
	S-CAM3	198,190	41.6	14	10	1883366	3	KU686199.1	NC_031906.1	[11]	2016
	S-CAM4	191,983	38.6	16	8	1883367	3	KU686201.1	NC_031900.1	[11]	2016
	S-CAM7	216,121	41.2	5	4	1883368	2	KU686212.1	NC_031927.1	[11]	2016
	S-CAM9	174,830	39	16	8	1883369	3	KU686206.1	NC_031922.1	[11]	2016
	S-CAM22	172,345	39.9	20	5	1883365	3	KU686209.1	NC_031903.1	[11]	2016
	S-WAM1	185,102	44.7	15	4	1815521	1	KU686210.1	NC_031944.1	[11]	2016
	S-WAM2	186,386	41.3	15	12	1815522	1	KU686211.1	NC_031935.1	[11]	2016
	S-CBWM1	139,069	51.6	3	36	2053653	1	MG450654.1	-	[67]	2017
	Bellamy	204,930	41.1	20	10	2023996	1	MF351863.1	-	-	2017
	S-H35	174,231	41.2	15	8	1983572	1	KY945241.1	-	-	2017
	S-B68	163,982	51.7	4	4	2545437	1	MK016664.1	-	-	2018
	S-B64	151,867	41.3	15	8	2163901	1	MH107246.1	-	-	2018
	S-PRM1	144,311	40.7	16	8	2100130	1	MH629685.1	-	-	2018
	S-T4	181,082	38.9	14	7	2268578	1	MH412654.1	-	-	2018
	S-E7	177,622	39.9	19	6	2484639	1	MH920640.1	-	-	2018
	B3	244,930	35.4	4	20	2674978	1	MN695334.1	-	-	2019
	B23	243,633	35.4	4	20	2674977	1	MN695335.1	-	-	2019
	S-RIM4	175,462	41.2	15	9	2530169	1	MK493321.1	-	-	2019
	S-B43	213,993	39.4	14	11	1340812	1	MN018232.1	-	-	2019
	S-B05	208,857	39.9	14	11	2484637	1	MK799832.1	-	[27]	2019
	**S-H34**	167,040	50.1	3	5	2718942	1	MT162467.2	-	This study	2020
	**S-N03**	167,069	50.1	4	1	2718943	1	MT162466.1	-	This study	2020
*Prochlorococcus*	P-SSM2	252,401	35.5	22	1	268746	2	AY939844.2	NC_006883.2	[45]	2005
	P-SSM4	178,249	36.7	19	0	268747	1	AY940168.2	NC_006884.2	[45]	2005
	P-SSM7	182,180	37.1	19	4	445688	1	GU071103.1	NC_015290.1	[45]	2005
	P-RSM4	176,428	37.6	19	3	444862	1	GU071099.1	NC_015283.1	[45]	2009
	P-HM1	181,044	37.8	17	0	445700	1	GU071101.1	NC_015280.1	[45]	2009
	P-HM2	183,806	38.1	15	0	445696	1	GU075905.1	NC_015284.1	[45]	2009
	P-RSM1	177,211	40.2	18	2	536444	1	HQ634175.1	NC_021071.1	-	2010
	P-RSM3	178,750	36.7	17	0	536446	1	HQ634176.1	-	-	2010
	P-RSM6	192,497	39.3	18	3	929832	1	HQ634193.1	NC_020855.1	-	2010
	P-SSM3	179,063	36.7	16	0	536453	1	HQ337021.1	NC_021559.1	-	2010
	P-SSM5	252,013	35.5	19	1	536454	1	HQ632825.1	-	-	2010
	MED4–213	180,977	37.8	15	0	889956	1	HQ634174.1	NC_020845.1	-	2010
	P-TIM40	188,632	40.7	17	1	1589733	1	KP211958.1	NC_028663.1	-	2014
	P-TIM68	197,361	34.3	14	0	1542477	1	KM359505.1	NC_028955.1	[68]	2014

**Table 2:** Spearman’s rank correlation coefficient between genome size, G + C content and AMGs. *: *p* < 0.05; **: *p* < 0.01; *n* = 81.

		Genome Size	GC%	AMGs
**Genome size**	Correlation coefficient	1	−0.340 **	0.001
	Sig.(*p*-value)	-	0.002	0.992
**GC%**	Correlation coefficient	−0.340 **	1	−0.272 *
	Sig.(*p*-value)	0.002	-	0.014
**AMGs**	Correlation coefficient	0.001	−0.272 *	1
	Sig.(*p*-value)	0.992	0.014	-

**Table 3:** tRNA-related information obtained by tRNA scan-SE.

Sequence Name	Position	Length (nt)	tRNA Type	Anticodon	Isotype Model	Isotype Score
*Synechococcus* phage S-N03						
tRNA1	101128–101057	72	Asn	GTT	Ans	98.7
*Synechococcus* phage S-H34						
tRNA1	157447–157366	82	Tyr	GTA	Tyr	70.1
tRNA2	155156–155085	72	Asn	GTT	Ans	98.7
tRNA3	155081–155009	73	Asp	GTC	Asp	66.5
tRNA4	154982–154911	72	Asn	GTT	Ans	98.7
tRNA5	154812–154741	72	Val	TAC	Phe	77.6

**Table 4:** Information of cyanophages with AMG content less than 10.

Phage	AMGs	Genome Size (kb)	GC (%)	Isolation Location	Host Name (*Syn*)	Host Isolation	Accession
S-N03	3	167,069	50.1	Yellow Sea, China	MW02	estuary	MT162466
S-H34	3	167,040	50.1	Yellow Sea, China	MW02	estuary	MT162467.2
S-B68	4	163,982	51.7	Bohai Sea, China	WH7803	marine	MK016664.1
S-CBWM1	4	139,069	51.6	Chesapeake Bay, USA	CBW1002	estuary	MG450654.1
S-CAM7	5	216,121	41.2	Crystal Cove, CA	WH7803	marine	NC_031927.1
S-CRM01	7	178,563	39.7	Copco Reservoir, Klamath River, CA	LC16	freshwater	NC_015569.1

## Supplementary Materials

The following are available online at https://www.mdpi.com/1999-4915/12/8/800/s1, Figure S1: Transmission electron micrographs of *Synechococcus* phages. (A) Cyanophage S-N03. (B) Cyanophage S-H34. Table S1: Infectivity of phage S-N03 and S-H34 against nine *Synechococcus* strains. Table S2: Statistics of *Prochlorococcus* and *Synechococcus* cyanomyoviruses that have uploaded the complete genome to the NCBI database by 2020. Table S3: Predicted ORFs in the S-N03 genome with homologues in the non-redundant database. Table S4: Predicted ORFs in the S-H34 genome with homologues in the non-redundant database.
