# Supplementary material for: Genome Analysis of Two Novel Synechococcus Phages That Lack Common Auxiliary Metabolic Genes: Possible Reasons and Ecological Insights by Comparative Analysis of Cyanomyoviruses
Source: Viruses. 2020 Jul 25;12(8):800. doi: 10.3390/v12080800 (PMC7472177; doi:10.3390/v12080800)
Supplement: Supplementary file 1 [file viruses-12-00800-s001.zip › Supplementary Materials Table S1.pdf]

Supplementary Materials Table S1. Infectivity of phage S-N03 and S-H34 against nine *Synechococcus* strains.

| | phylogenetic clade | pigment type[1] | phenotype | isolation site | S-N03 infect[2] | S-H34 infect | Ref. |
|---|---|---|---|---|---|---|---|
| MW02 | S5.1-clade IX | 2 | PE-type | HK | + | + | [1] |
| WH7803 | S5.1-clade V | 3a | PE-type | HK | + | + | [1] |
| WH8102 | S5.1-clade II | 3c | PE-type | HK | + | + | [1] |
| LTWRED | S5.2 | 2 | PE-type | HK | + | + | PC |
| LTWGREEN | S5.2 | 1 | PC-type | HK | - | - | PC |
| MW03 | S5.1-clade VIII | 1 | PC-type | HK | - | - | [1] |
| PCC7002 | - | 1 | PC-type | HK | - | - | PC |
| PSHK05 | - | 1 | PC-type | HK | - | - | PC |
| CCMP1333 | - | 1 | PC-type | HK | - | - | PC |

[1] 1: phycocyanin-only (PC-type), 2: phycoerythrin-only (PE-type), 3a: low-PUB: PEB (PE-type), 3c: high-PUB: PEB (PE-type) (PUB: phycourobilin, PEB: phycoerythrobilin)

[2] "+" indicates that the host can be infected, "-" indicates that the host cannot be infected. PC: personal communication

**References**

[1] Xia, X.; Vidyarathna, N.K.; Palenik, B.; Lee, P.; Liu, H. Comparison of the seasonal variations of *Synechococcus* Assemblage structures in estuarine waters and coastal waters of Hong Kong. *Applied & Environmental Microbiology* **2015**, *81(21), 7644*. http://dx.doi.org/10.1128/AEM.01895-15
