# Supplementary material for: Genome Analysis of Two Novel Synechococcus Phages That Lack Common Auxiliary Metabolic Genes: Possible Reasons and Ecological Insights by Comparative Analysis of Cyanomyoviruses
Source: Viruses. 2020 Jul 25;12(8):800. doi: 10.3390/v12080800 (PMC7472177; doi:10.3390/v12080800)
Supplement: Supplementary file 1 [file viruses-12-00800-s001.zip › Supplementary Materials Table S2.pdf]

Supplementary Materials Table S2. Statistics of *Prochlorococcus* and *Synechococcus* cyanomyoviruses that have uploaded the complete genome to the NCBI database by 2020. "*Syn*" refers to *Synechococcus* and "*Pro*" refers to *Prochlorococcus*. The phage name and accession number of the selected 81 representative cyanophages are shown in bold font.

Host	Phage name	Taxonomy ID	No. isolates	Sequence name	Accession	RefSeq Accession	Ref.	Submission date
Pro	**P-SSM2**	268746	2	Prochlorococcus phage P-SSM2, complete genome	**AY939844.2**	**NC_006883.2**	[1]	2005
				Prochlorococcus phage P-SSM2, complete genome	GU071092.1	-	-	2005
Pro	**P-SSM7**	445688	1	Prochlorococcus phage P-SSM7, complete genome	**GU071103.1**	**NC_015290.1**	[1]	2005
Pro	**P-SSM4**	268747	1	Prochlorococcus phage P-SSM4, complete genome	**AY940168.2**	**NC_006884.2**	[1]	2005
Pro	**P-RSM4**	444862	1	Prochlorococcus phage P-RSM4, complete genome	**GU071099.1**	**NC_015283.1**	[1]	2009
Pro	**P-HM2**	445696	1	Prochlorococcus phage P-HM2, complete genome	**GU075905.1**	**NC_015284.1**	[1]	2009
Pro	**P-HM1**	445700	1	Prochlorococcus phage P-HM1, complete genome	**GU071101.1**	**NC_015280.1**	[1]	2009
Pro	**P-RSM1**	536444	1	Cyanophage P-RSM1 genomic sequence	**HQ634175.1**	**NC_021071.1**	-	2010
Pro	**P-RSM6**	929832	1	Cyanophage P-RSM6 genomic sequence	**HQ634193.1**	**NC_020855.1**	-	2010
Pro	**P-RSM3**	536446	1	Cyanophage P-RSM3 genomic sequence	**HQ634176.1**	-	-	2010
Pro	**P-SSM5**	536454	1	Prochlorococcus phage P-SSM5 genomic sequence	**HQ632825.1**	-	-	2010
Pro	**P-SSM3**	536453	1	Prochlorococcus phage P-SSM3 genomic sequence	**HQ337021.1**	**NC_021559.1**	-	2010
Pro	**MED4-213**	889956	1	Cyanophage MED4-213, complete genome	**HQ634174.1**	**NC_020845.1**	-	2010
Pro	**P-TIM68**	1542477	1	Prochlorococcus phage P-TIM68, complete genome	**KM359505.1**	**NC_028955.1**	[2]	2014
Pro	**P-TIM40**	1589733	1	Cyanophage P-TIM40, complete genome	**KP211958.1**	**NC_028663.1**	-	2014
Syn	**S-RIM2 R1_1999**	869662	1	Synechococcus phage S-RIM2 R1_1999, complete genome	**HQ317292.1**	**NC_020859.1**	[3]	2016
Syn	S-RIM2 R21_2007	869661	1	Synechococcus phage S-RIM2 R21_2007, complete genome	HQ317290.1	-	[3]	2016
Syn	S-RIM2 R9_2006	869663	1	Synechococcus phage S-RIM2 R9_2006, complete genome	HQ317291.1	-	[3]	2016
Syn	S-RIM2	687800	59	Synechococcus phage S-RIM2 isolate Fa_02_0709, complete genome	KX349226.1	-	[3]	2016
				Synechococcus phage S-RIM2 isolate Fa_10_0709, complete genome	KX349227.1	-	[3]	2016
				Synechococcus phage S-RIM2 isolate Fa_24_0709, complete genome	KX349228.1	-	[3]	2016
				Synechococcus phage S-RIM2 isolate LIS_01_1010, complete genome	KX349229.1	-	[3]	2016
				Synechococcus phage S-RIM2 isolate LIS_02_1013, complete genome	KX349230.1	-	[3]	2016
				Synechococcus phage S-RIM2 isolate LIS_06_1010, complete genome	KX349231.1	-	[3]	2016
				Synechococcus phage S-RIM2 isolate LIS_09_1010, complete genome	KX349232.1	-	[3]	2016
				Synechococcus phage S-RIM2 isolate LIS_11_1010, complete genome	KX349233.1	-	[3]	2016
				Synechococcus phage S-RIM2 isolate LIS_12_1010, complete genome	KX349234.1	-	[3]	2016
				Synechococcus phage S-RIM2 isolate LIS_14_1013, complete genome	KX349235.1	-	[3]	2016
				Synechococcus phage S-RIM2 isolate NJ_05_1013, complete genome	KX349236.1	-	[3]	2016
				Synechococcus phage S-RIM2 isolate Np_01_0709, complete genome	KX349237.1	-	[3]	2016
				Synechococcus phage S-RIM2 isolate Np_01_1112, complete genome	KX349238.1	-	[3]	2016
				Synechococcus phage S-RIM2 isolate Np_03_0709, complete genome	KX349239.1	-	[3]	2016
				Synechococcus phage S-RIM2 isolate Np_03_1112, complete genome	KX349240.1	-	[3]	2016
				Synechococcus phage S-RIM2 isolate Np_04_1112, complete genome	KX349241.1	-	[3]	2016
				Synechococcus phage S-RIM2 isolate Np_06_0912, complete genome	KX349242.1	-	[3]	2016
				Synechococcus phage S-RIM2 isolate Np_11_1112, complete genome	KX349243.1	-	[3]	2016

	Synechococcus phage S-RIM2 isolate Np_12_0912, complete genome	KX349244.1	-	[3]	2016
	Synechococcus phage S-RIM2 isolate Np_14_0912, complete genome	KX349245.1	-	[3]	2016
	Synechococcus phage S-RIM2 isolate Np_15_0709, complete genome	KX349246.1	-	[3]	2016
	Synechococcus phage S-RIM2 isolate Np_15_1112, complete genome	KX349247.1	-	[3]	2016
	Synechococcus phage S-RIM2 isolate Np_19_1112, complete genome	KX349248.1	-	[3]	2016
	Synechococcus phage S-RIM2 isolate Np_20_0912, complete genome	KX349249.1	-	[3]	2016
	Synechococcus phage S-RIM2 isolate Np_23_1112, complete genome	KX349250.1	-	[3]	2016
	Synechococcus phage S-RIM2 isolate Np_24_1112, complete genome	KX349251.1	-	[3]	2016
	Synechococcus phage S-RIM2 isolate Np_31_1112, complete genome	KX349252.1	-	[3]	2016
	Synechococcus phage S-RIM2 isolate Np_33_0912, complete genome	KX349253.1	-	[3]	2016
	Synechococcus phage S-RIM2 isolate Np_36_1112, complete genome	KX349254.1	-	[3]	2016
	Synechococcus phage S-RIM2 isolate RW_01_0709, complete genome	KX349255.1	-	[3]	2016
	Synechococcus phage S-RIM2 isolate RW_02_0113, complete genome	KX349256.1	-	[3]	2016
	Synechococcus phage S-RIM2 isolate RW_02_0709, complete genome	KX349257.1	-	[3]	2016
	Synechococcus phage S-RIM2 isolate RW_03_0709, complete genome	KX349258.1	-	[3]	2016
	Synechococcus phage S-RIM2 isolate RW_08_1112, complete genome	KX349259.1	-	[3]	2016
	Synechococcus phage S-RIM2 isolate RW_11_0905, complete genome	KX349260.1	-	[3]	2016
	Synechococcus phage S-RIM2 isolate RW_12_0113, complete genome	KX349261.1	-	[3]	2016
	Synechococcus phage S-RIM2 isolate RW_12_0709, complete genome	KX349262.1	-	[3]	2016
	Synechococcus phage S-RIM2 isolate RW_14_1112, complete genome	KX349263.1	-	[3]	2016
	Synechococcus phage S-RIM2 isolate RW_16_0905, complete genome	KX349264.1	-	[3]	2016
	Synechococcus phage S-RIM2 isolate RW_17_0113, complete genome	KX349265.1	-	[3]	2016
	Synechococcus phage S-RIM2 isolate RW_26_0905, complete genome	KX349266.1	-	[3]	2016
	Synechococcus phage S-RIM2 isolate RW_29_1112, complete genome	KX349267.1	-	[3]	2016
	Synechococcus phage S-RIM2 isolate RW_30_0905, complete genome	KX349268.1	-	[3]	2016
	Synechococcus phage S-RIM2 isolate RW_34_0905, complete genome	KX349269.1	-	[3]	2016
	Synechococcus phage S-RIM2 isolate RW_40_1112, complete genome	KX349270.1	-	[3]	2016
	Synechococcus phage S-RIM2 isolate Sn_25_0709, complete genome	KX349271.1	-	[3]	2016
	Synechococcus phage S-RIM2 isolate W1_01_0709, complete genome	KX349272.1	-	[3]	2016
	Synechococcus phage S-RIM2 isolate W1_01_0910, complete genome	KX349273.1	-	[3]	2016
	Synechococcus phage S-RIM2 isolate W1_03_0709, complete genome	KX349274.1	-	[3]	2016
	Synechococcus phage S-RIM2 isolate W1_08_0709, complete genome	KX349275.1	-	[3]	2016
	Synechococcus phage S-RIM2 isolate W1_09_0709, complete genome	KX349276.1	-	[3]	2016
	Synechococcus phage S-RIM2 isolate W1_12_0909, complete genome	KX349277.1	-	[3]	2016
	Synechococcus phage S-RIM2 isolate W1_13_0709, complete genome	KX349278.1	-	[3]	2016
	Synechococcus phage S-RIM2 isolate W1_16_0709, complete genome	KX349279.1	-	[3]	2016
	Synechococcus phage S-RIM2 isolate W2_02_0709, complete genome	KX349280.1	-	[3]	2016
	Synechococcus phage S-RIM2 isolate W2_13_0910, complete genome	KX349281.1	-	[3]	2016
	Synechococcus phage S-RIM2 isolate W2_14_0910, complete genome	KX349282.1	-	[3]	2016
	Synechococcus phage S-RIM2 isolate W2_32_0910, complete genome	KX349283.1	-	[3]	2016
	Synechococcus phage S-RIM2 isolate W2_39_0910, complete genome	KX349284.1	-	[3]	2016

Syn	**ACG-2014d**	1493509	45	Synechococcus phage ACG-2014d isolate Syn7803C102, complete genome	**KJ019136.1**	**NC_026923.1**	[4]	2013
				Synechococcus phage ACG-2014d isolate Syn7803C45, complete genome	KJ019028.1	-	[4]	2013
				Synechococcus phage ACG-2014d isolate Syn7803C46, complete genome	KJ019029.1	-	[4]	2013
				Synechococcus phage ACG-2014d isolate Syn7803C48, complete genome	KJ019031.1	-	[4]	2013
				Synechococcus phage ACG-2014d isolate Syn7803C49, complete genome	KJ019032.1	-	[4]	2013
				Synechococcus phage ACG-2014d isolate Syn7803C54, complete genome	KJ019034.1	-	[4]	2013
				Synechococcus phage ACG-2014d isolate Syn7803C55, complete genome	KJ019035.1	-	[4]	2013
				Synechococcus phage ACG-2014d isolate Syn7803C57, complete genome	KJ019036.1	-	[4]	2013
				Synechococcus phage ACG-2014d isolate Syn7803C72, complete genome	KJ019046.1	-	[4]	2013
				Synechococcus phage ACG-2014d isolate Syn7803C73, complete genome	KJ019047.1	-	[4]	2013
				Synechococcus phage ACG-2014d isolate Syn7803C75, complete genome	KJ019048.1	-	[4]	2013
				Synechococcus phage ACG-2014d isolate Syn7803C77, complete genome	KJ019050.1	-	[4]	2013
				Synechococcus phage ACG-2014d isolate Syn7803C89, complete genome	KJ019057.1	-	[4]	2013
				Synechococcus phage ACG-2014d isolate Syn7803C93, complete genome	KJ019062.1	-	[4]	2013
				Synechococcus phage ACG-2014d isolate Syn7803US104, complete genome	KJ019070.1	-	[4]	2013
				Synechococcus phage ACG-2014d isolate Syn7803US108, complete genome	KJ019072.1	-	[4]	2013
				Synechococcus phage ACG-2014d isolate Syn7803US109, complete genome	KJ019073.1	-	[4]	2013
				Synechococcus phage ACG-2014d isolate Syn7803US110, complete genome	KJ019074.1	-	[4]	2013
				Synechococcus phage ACG-2014d isolate Syn7803US111, complete genome	KJ019075.1	-	[4]	2013
				Synechococcus phage ACG-2014d isolate Syn7803US113, complete genome	KJ019077.1	-	[4]	2013
				Synechococcus phage ACG-2014d isolate Syn7803US114, complete genome	KJ019078.1	-	[4]	2013
				Synechococcus phage ACG-2014d isolate Syn7803US115, complete genome	KJ019079.1	-	[4]	2013
				Synechococcus phage ACG-2014d isolate Syn7803US116, complete genome	KJ019080.1	-	[4]	2013
				Synechococcus phage ACG-2014d isolate Syn7803US122, complete genome	KJ019083.1	-	[4]	2013
				Synechococcus phage ACG-2014d isolate Syn7803US59, complete genome	KJ019112.1	-	[4]	2013
				Synechococcus phage ACG-2014d isolate Syn7803US5, complete genome	KJ019113.1	-	[4]	2013
				Synechococcus phage ACG-2014d isolate Syn7803US61, complete genome	KJ019115.1	-	[4]	2013
				Synechococcus phage ACG-2014d isolate Syn7803US63, complete genome	KJ019117.1	-	[4]	2013
				Synechococcus phage ACG-2014d isolate Syn7803US64, complete genome	KJ019118.1	-	[4]	2013
				Synechococcus phage ACG-2014d isolate Syn7803US65, complete genome	KJ019119.1	-	[4]	2013
				Synechococcus phage ACG-2014d isolate Syn7803US71, complete genome	KJ019120.1	-	[4]	2013
				Synechococcus phage ACG-2014d isolate Syn7803US78, complete genome	KJ019121.1	-	[4]	2013
				Synechococcus phage ACG-2014d isolate Syn7803US80, complete genome	KJ019124.1	-	[4]	2013
				Synechococcus phage ACG-2014d isolate Syn7803US82, complete genome	KJ019125.1	-	[4]	2013
				Synechococcus phage ACG-2014d isolate Syn7803US83, complete genome	KJ019126.1	-	[4]	2013
				Synechococcus phage ACG-2014d isolate Syn7803US85, complete genome	KJ019127.1	-	[4]	2013
				Synechococcus phage ACG-2014d isolate Syn7803US89, complete genome	KJ019129.1	-	[4]	2013
				Synechococcus phage ACG-2014d isolate Syn7803US94, complete genome	KJ019130.1	-	[4]	2013
				Synechococcus phage ACG-2014d isolate Syn7803US95, complete genome	KJ019131.1	-	[4]	2013
				Synechococcus phage ACG-2014d isolate Syn7803C108, complete genome	KJ019139.1	-	[4]	2013
				Synechococcus phage ACG-2014d isolate Syn7803C109, complete genome	KJ019140.1	-	[4]	2013

				Synechococcus phage ACG-2014d isolate Syn7803C35, complete genome	KJ019160.1	-	[4]	2013
				Synechococcus phage ACG-2014d isolate Syn7803C37, complete genome	KJ019162.1	-	[4]	2013
				Synechococcus phage ACG-2014d isolate Syn7803C39, complete genome	KJ019164.1	-	[4]	2013
				Synechococcus phage ACG-2014d isolate Syn7803C40, complete genome	KJ019165.1	-	[4]	2013
Syn	**ACG-2014f**	1493511	41	Synechococcus phage ACG-2014f isolate Syn7803C90, complete genome	**KJ019059.1**	**NC_026927.1**	[4]	2013
				Synechococcus phage ACG-2014f isolate Syn7803C58, complete genome	KJ019037.1	-	[4]	2013
				Synechococcus phage ACG-2014f isolate Syn7803C6, complete genome	KJ019045.1	-	[4]	2013
				Synechococcus phage ACG-2014f isolate Syn7803C7, complete genome	KJ019052.1	-	[4]	2013
				Synechococcus phage ACG-2014f isolate Syn7803C80, complete genome	KJ019053.1	-	[4]	2013
				Synechococcus phage ACG-2014f isolate Syn7803C8, complete genome	KJ019058.1	-	[4]	2013
				Synechococcus phage ACG-2014f isolate Syn7803C9, complete genome	KJ019066.1	-	[4]	2013
				Synechococcus phage ACG-2014f isolate Syn7803US13, complete genome	KJ019085.1	-	[4]	2013
				Synechococcus phage ACG-2014f isolate Syn7803US17, complete genome	KJ019086.1	-	[4]	2013
				Synechococcus phage ACG-2014f isolate Syn7803US24, complete genome	KJ019090.1	-	[4]	2013
				Synechococcus phage ACG-2014f isolate Syn7803US26, complete genome	KJ019091.1	-	[4]	2013
				Synechococcus phage ACG-2014f isolate Syn7803US2, complete genome	KJ019092.1	-	[4]	2013
				Synechococcus phage ACG-2014f isolate Syn7803US30, complete genome	KJ019093.1	-	[4]	2013
				Synechococcus phage ACG-2014f isolate Syn7803US34, complete genome	KJ019095.1	-	[4]	2013
				Synechococcus phage ACG-2014f isolate Syn7803US36, complete genome	KJ019096.1	-	[4]	2013
				Synechococcus phage ACG-2014f isolate Syn7803US37, complete genome	KJ019097.1	-	[4]	2013
				Synechococcus phage ACG-2014f isolate Syn7803US39, complete genome	KJ019098.1	-	[4]	2013
				Synechococcus phage ACG-2014f isolate Syn7803US3, complete genome	KJ019099.1	-	[4]	2013
				Synechococcus phage ACG-2014f isolate Syn7803US40, complete genome	KJ019100.1	-	[4]	2013
				Synechococcus phage ACG-2014f isolate Syn7803US42, complete genome	KJ019101.1	-	[4]	2013
				Synechococcus phage ACG-2014f isolate Syn7803US43, complete genome	KJ019102.1	-	[4]	2013
				Synechococcus phage ACG-2014f isolate Syn7803US44, complete genome	KJ019103.1	-	[4]	2013
				Synechococcus phage ACG-2014f isolate Syn7803US4, complete genome	KJ019105.1	-	[4]	2013
				Synechococcus phage ACG-2014f isolate Syn7803US50, complete genome	KJ019106.1	-	[4]	2013
				Synechococcus phage ACG-2014f isolate Syn7803US52, complete genome	KJ019107.1	-	[4]	2013
				Synechococcus phage ACG-2014f isolate Syn7803US57, complete genome	KJ019111.1	-	[4]	2013
				Synechococcus phage ACG-2014f isolate Syn7803US7, complete genome	KJ019123.1	-	[4]	2013
				Synechococcus phage ACG-2014f isolate Syn7803C10, complete genome	KJ019141.1	-	[4]	2013
				Synechococcus phage ACG-2014f isolate Syn7803C11, complete genome	KJ019142.1	-	[4]	2013
				Synechococcus phage ACG-2014f isolate Syn7803C12, complete genome	KJ019143.1	-	[4]	2013
				Synechococcus phage ACG-2014f isolate Syn7803C14, complete genome	KJ019144.1	-	[4]	2013
				Synechococcus phage ACG-2014f isolate Syn7803C15, complete genome	KJ019145.1	-	[4]	2013
				Synechococcus phage ACG-2014f isolate Syn7803C16, complete genome	KJ019146.1	-	[4]	2013
				Synechococcus phage ACG-2014f isolate Syn7803C17, complete genome	KJ019147.1	-	[4]	2013
				Synechococcus phage ACG-2014f isolate Syn7803C19, complete genome	KJ019148.1	-	[4]	2013
				Synechococcus phage ACG-2014f isolate Syn7803C21, complete genome	KJ019149.1	-	[4]	2013
				Synechococcus phage ACG-2014f isolate Syn7803C22, complete genome	KJ019150.1	-	[4]	2013

				Synechococcus phage ACG-2014f isolate Syn7803C24, complete genome	KJ019151.1	-	[4]	2013
				Synechococcus phage ACG-2014f isolate Syn7803C25, complete genome	KJ019152.1	-	[4]	2013
				Synechococcus phage ACG-2014f isolate Syn7803C29, complete genome	KJ019155.1	-	[4]	2013
				Synechococcus phage ACG-2014f isolate Syn7803C34, complete genome	KJ019159.1	-	[4]	2013
Syn	**ACG-2014a**	1493507	24	Synechococcus phage ACG-2014a isolate Syn7803C42, complete genome	**KJ019026.1**	-	[4]	2013
				Synechococcus phage ACG-2014a isolate Syn7803C47, complete genome	KJ019030.1	-	[4]	2013
				Synechococcus phage ACG-2014a isolate Syn7803C53, complete genome	KJ019033.1	-	[4]	2013
				Synechococcus phage ACG-2014a isolate Syn7803C59, complete genome	KJ019038.1	-	[4]	2013
				Synechococcus phage ACG-2014a isolate Syn7803C60, complete genome	KJ019039.1	-	[4]	2013
				Synechococcus phage ACG-2014a isolate Syn7803C86, complete genome	KJ019055.1	-	[4]	2013
				Synechococcus phage ACG-2014a isolate Syn7803C99, complete genome	KJ019065.1	-	[4]	2013
				Synechococcus phage ACG-2014a isolate Syn7803US101, complete genome	KJ019067.1	-	[4]	2013
				Synechococcus phage ACG-2014a isolate Syn7803US102, complete genome	KJ019068.1	-	[4]	2013
				Synechococcus phage ACG-2014a isolate Syn7803US112, complete genome	KJ019076.1	-	[4]	2013
				Synechococcus phage ACG-2014a isolate Syn7803US117, complete genome	KJ019081.1	-	[4]	2013
				Synechococcus phage ACG-2014a isolate Syn7803US123, complete genome	KJ019084.1	-	[4]	2013
				Synechococcus phage ACG-2014a isolate Syn7803US19, complete genome	KJ019087.1	-	[4]	2013
				Synechococcus phage ACG-2014a isolate Syn7803US1, complete genome	KJ019088.1	-	[4]	2013
				Synechococcus phage ACG-2014a isolate Syn7803US60, complete genome	KJ019114.1	-	[4]	2013
				Synechococcus phage ACG-2014a isolate Syn7803US62, complete genome	KJ019116.1	-	[4]	2013
				Synechococcus phage ACG-2014a isolate Syn7803US79, complete genome	KJ019122.1	-	[4]	2013
				Synechococcus phage ACG-2014a isolate Syn7803C101, complete genome	KJ019135.1	-	[4]	2013
				Synechococcus phage ACG-2014a isolate Syn7803C104, complete genome	KJ019137.1	-	[4]	2013
				Synechococcus phage ACG-2014a isolate Syn7803C107, complete genome	KJ019138.1	-	[4]	2013
				Synechococcus phage ACG-2014a isolate Syn7803C26, complete genome	KJ019153.1	-	[4]	2013
				Synechococcus phage ACG-2014a isolate Syn7803C31, complete genome	KJ019157.1	-	[4]	2013
				Synechococcus phage ACG-2014a isolate Syn7803C33, complete genome	KJ019158.1	-	[4]	2013
				Synechococcus phage ACG-2014a isolate Syn7803C38, complete genome	KJ019163.1	-	[4]	2013
Syn	**S-RIM12**	1278402	21	Cyanophage S-RIM12 isolate Np_14_0310, complete genome	**KX349307.1**	-	[3]	2016
				Cyanophage S-RIM12 isolate Np_15_0310, complete genome	KX349308.1	-	[3]	2016
				Cyanophage S-RIM12 isolate Np_22_1112, complete genome	KX349309.1	-	[3]	2016
				Cyanophage S-RIM12 isolate RW_01_0310, complete genome	KX349310.1	-	[3]	2016
				Cyanophage S-RIM12 isolate RW_04_0310, complete genome	KX349311.1	-	[3]	2016
				Cyanophage S-RIM12 isolate RW_04_0709, complete genome	KX349312.1	-	[3]	2016
				Cyanophage S-RIM12 isolate RW_06_0310, complete genome	KX349313.1	-	[3]	2016
				Cyanophage S-RIM12 isolate RW_07_1112, complete genome	KX349314.1	-	[3]	2016
				Cyanophage S-RIM12 isolate RW_14_0101, complete genome	KX349315.1	-	[3]	2016
				Cyanophage S-RIM12 isolate RW_22_0110, complete genome	KX349316.1	-	[3]	2016
				Cyanophage S-RIM12 isolate RW_25_0210, complete genome	KX349317.1	-	[3]	2016
				Cyanophage S-RIM12 isolate RW_27_0310, complete genome	KX349318.1	-	[3]	2016
				Cyanophage S-RIM12 isolate RW_28_1109, complete genome	KX349319.1	-	[3]	2016

				Cyanophage S-RIM12 isolate RW_29_1109, complete genome	KX349320.1	-	[3]	2016
				Cyanophage S-RIM12 isolate Sn_07_0910, complete genome	KX349321.1	-	[3]	2016
				Cyanophage S-RIM12 isolate Sn_31_0910, complete genome	KX349322.1	-	[3]	2016
				Cyanophage S-RIM12 isolate W1_08_0910, complete genome	KX349323.1	-	[3]	2016
				Cyanophage S-RIM12 isolate W1_12_0610, complete genome	KX349324.1	-	[3]	2016
				Cyanophage S-RIM12 isolate W1_24_0910, complete genome	KX349325.1	-	[3]	2016
				Cyanophage S-RIM12 isolate WH_05_0310, complete genome	KX349326.1	-	[3]	2016
				Cyanophage S-RIM12 isolate WH_07_0310, complete genome	KX349327.1	-	[3]	2016
Syn	**ACG-2014b**	1493508	18	Synechococcus phage ACG-2014b isolate Syn7803C100, complete genome	**KJ019134.1**	**NC_027130.1**	[4]	2013
				Synechococcus phage ACG-2014b isolate Syn7803C61, complete genome	KJ019040.1	-	[4]	2013
				Synechococcus phage ACG-2014b isolate Syn7803C66, complete genome	KJ019041.1	-	[4]	2013
				Synechococcus phage ACG-2014b isolate Syn7803C67, complete genome	KJ019042.1	-	[4]	2013
				Synechococcus phage ACG-2014b isolate Syn7803C68, complete genome	KJ019043.1	-	[4]	2013
				Synechococcus phage ACG-2014b isolate Syn7803C69, complete genome	KJ019044.1	-	[4]	2013
				Synechococcus phage ACG-2014b isolate Syn7803C76, complete genome	KJ019049.1	-	[4]	2013
				Synechococcus phage ACG-2014b isolate Syn7803C78, complete genome	KJ019051.1	-	[4]	2013
				Synechococcus phage ACG-2014b isolate Syn7803C91, complete genome	KJ019060.1	-	[4]	2013
				Synechococcus phage ACG-2014b isolate Syn7803C92, complete genome	KJ019061.1	-	[4]	2013
				Synechococcus phage ACG-2014b isolate Syn7803US49, complete genome	KJ019104.1	-	[4]	2013
				Synechococcus phage ACG-2014b isolate Syn7803US53, complete genome	KJ019108.1	-	[4]	2013
				Synechococcus phage ACG-2014b isolate Syn7803US54, complete genome	KJ019109.1	-	[4]	2013
				Synechococcus phage ACG-2014b isolate Syn7803US56, complete genome	KJ019110.1	-	[4]	2013
				Synechococcus phage ACG-2014b isolate Syn9311C1, complete genome	KJ019132.1	-	[4]	2013
				Synechococcus phage ACG-2014b isolate Syn9311C4, complete genome	KJ019133.1	-	[4]	2013
				Synechococcus phage ACG-2014b isolate Syn7803C28, complete genome	KJ019154.1	-	[4]	2013
				Synechococcus phage ACG-2014b isolate Syn7803C36, complete genome	KJ019161.1	-	[4]	2013
Syn	**S-RIM8 A.HR1**	869724	1	Synechococcus phage S-RIM8 A.HR1, complete genome	**JF974288.1**	**NC_020486.1**	[5]	2010
Syn	S-RIM8 A.HR5	869726	1	Synechococcus phage S-RIM8 A.HR5, complete genome	HQ317385.1	-	[5]	2010
Syn	S-RIM8 A.HR3	869725	1	Synechococcus phage S-RIM8 A.HR3, complete genome	JF974289.1	-	[5]	2010
Syn	S-RIM8	756278	10	Synechococcus phage S-RIM8 isolate RW_01_0115_WH8101, complete genome	MK493322.1	-	-	2019
				Synechococcus phage S-RIM8 isolate RW_03_0617, complete genome	MK493323.1	-	-	2019
				Synechococcus phage S-RIM8 isolate RW_22_0214, complete genome	MK493324.1	-	-	2019
				Synechococcus phage S-RIM8 isolate RW_62_0316, complete genome	MK493325.1	-	-	2019
				Synechococcus phage S-RIM8 isolate RW_01_0212_WH8101, complete genome	KX349285.1	-	[3]	2016
				Synechococcus phage S-RIM8 isolate RW_03_0807_WH8101, complete genome	KX349286.1	-	[3]	2016
				Synechococcus phage S-RIM8 isolate RW_06_0613, complete genome	KX349287.1	-	[3]	2016
				Synechococcus phage S-RIM8 isolate RW_08_0711, complete genome	KX349288.1	-	[3]	2016
				Synechococcus phage S-RIM8 isolate RW_22_0300, complete genome	KX349289.1	-	[3]	2016
				Synechococcus phage S-RIM8 isolate RW_25_1112, complete genome	KX349290.1	-	[3]	2016
Syn	**S-RIM14**	1278423	9	Cyanophage S-RIM14 isolate LIS_02_1110, complete genome	**KX349298.1**	-	[3]	2016
				Cyanophage S-RIM14 isolate LIS_22_0610, complete genome	KX349299.1	-	[3]	2016

				Cyanophage S-RIM14 isolate Np_11_1211, complete genome	KX349300.1	-	[3]	2016
				Cyanophage S-RIM14 isolate Np_45_0711, complete genome	KX349301.1	-	[3]	2016
				Cyanophage S-RIM14 isolate RW_03_0110, complete genome	KX349302.1	-	[3]	2016
				Cyanophage S-RIM14 isolate Sn_11_0110, complete genome	KX349303.1	-	[3]	2016
				Cyanophage S-RIM14 isolate Sn_18_0910, complete genome	KX349304.1	-	[3]	2016
				Cyanophage S-RIM14 isolate Sn_23_0910, complete genome	KX349305.1	-	[3]	2016
				Cyanophage S-RIM14 isolate W1_23_0910, complete genome	KX349306.1	-	[3]	2016
Syn	**S-RIM44**	1278485	8	Cyanophage S-RIM44 isolate ES_42_0910, complete genome	**KX349291.1**	-	[3]	2016
				Cyanophage S-RIM44 isolate Np_05_0604, complete genome	KX349292.1	-	[3]	2016
				Cyanophage S-RIM44 isolate Np_20_0711, complete genome	KX349293.1	-	[3]	2016
				Cyanophage S-RIM44 isolate Np_42_0711, complete genome	KX349294.1	-	[3]	2016
				Cyanophage S-RIM44 isolate Sn_08_0709, complete genome	KX349295.1	-	[3]	2016
				Cyanophage S-RIM44 isolate Sn_13_0910, complete genome	KX349296.1	-	[3]	2016
				Cyanophage S-RIM44 isolate W2_10_0709, complete genome	KX349297.1	-	[3]	2016
				Cyanophage S-RIM44 isolate W2_07_0710, complete genome	KU594607.1	-	[3]	2016
Syn	**S-CAM1**	754037	6	Synechococcus phage S-CAM1 genomic sequence	**HQ634177.1**	**NC_020837.1**	-	2010
				Synechococcus phage S-CAM1 isolate 0309SB33, complete genome	KU686192.1	-	[6]	2016
				Synechococcus phage S-CAM1 isolate 0310NB17, complete genome	KU686193.1	-	[6]	2016
				Synechococcus phage S-CAM1 isolate 0809CC03, complete genome	KU686194.1	-	[6]	2016
				Synechococcus phage S-CAM1 isolate 0810SB17, complete genome	KU686195.1	-	[6]	2016
				Synechococcus phage S-CAM1 isolate 0910CC29, complete genome	KU686196.1	-	[6]	2016
Syn	**ACG-2014c**	1079998	5	Synechococcus phage S-MbCM6, complete genome	**JN371768.1**	**NC_019444.1**	[7]	2011
				Synechococcus phage ACG-2014c isolate Syn7803C43, complete genome	KJ019027.1	-	[4]	2013
				Synechococcus phage ACG-2014c isolate Syn7803C97, complete genome	KJ019063.1	-	[4]	2013
				Synechococcus phage ACG-2014c isolate Syn7803C98, complete genome	KJ019064.1	-	[4]	2013
				Synechococcus phage ACG-2014c isolate Syn7803US88, complete genome	KJ019128.1	-	[4]	2013
Syn	**ACG-2014e**	1493510	3	Synechococcus phage ACG-2014e isolate Syn7803C2, complete genome	**KJ019156.1**	**NC_026928.1**	[4]	2013
				Synechococcus phage ACG-2014e isolate Syn7803C85, complete genome	KJ019054.1	-	[4]	2013
				Synechococcus phage ACG-2014e isolate Syn7803US33, complete genome	KJ019094.1	-	[4]	2013
Syn	**S-CAM3**	1883366	3	Synechococcus phage S-CAM3 isolate 1010CC42, complete genome	**KU686199.1**	**NC_031906.1**	[6]	2016
				Synechococcus phage S-CAM3 isolate 0808SB25, complete genome	KU686197.1	-	[6]	2016
				Synechococcus phage S-CAM3 isolate 0910TB04, complete genome	KU686198.1	-	[6]	2016
Syn	**S-CAM9**	1883369	3	Synechococcus phage S-CAM9 isolate 1109NB16, complete genome	**KU686206.1**	**NC_031922.1**	[6]	2016
				Synechococcus phage S-CAM9 isolate 0808SB05, complete genome	KU686204.1	-	[6]	2016
				Synechococcus phage S-CAM9 isolate 0908SB82, complete genome	KU686205.1	-	[6]	2016
Syn	**S-CAM22**	1883365	3	Synechococcus phage S-CAM22 isolate 1209TA19, complete genome	**KU686209.1**	**NC_031903.1**	[6]	2016
				Synechococcus phage S-CAM22 isolate 0210CC35, complete genome	KU686207.1	-	[6]	2016
				Synechococcus phage S-CAM22 isolate 0310NB44, complete genome	KU686208.1	-	[6]	2016
Syn	**S-CAM4**	1883367	3	Synechococcus phage S-CAM4 isolate 0809SB33, complete genome	**KU686201.1**	**NC_031900.1**	[6]	2016
				Synechococcus phage S-CAM4 isolate 0309CC44, complete genome	KU686200.1	-	[6]	2016
				Synechococcus phage S-CAM4 isolate 1010NB23, complete genome	KU686202.1	-	[6]	2016

Syn	**S-PM2**	238854	2	Synechococcus phage S-PM2, complete genome	**AJ630128.1**	**NC_006820.1**	[8]	2004
				Synechococcus phage S-PM2 spontaneous deletion mutant, complete genome	LN828717.1	-	[9]	2015
Syn	**S-CAM8**	754038	2	Synechococcus phage S-CAM8 strain S-CAM8 06008BI06, complete genome	**HQ634178.1**	**NC_021530.1**	-	2010
				Synechococcus phage S-CAM8 isolate 0810PA29, complete genome	KU686203.1	-	[6]	2016
Syn	**ACG-2014j**	1493514	2	Synechococcus phage ACG-2014j isolate Syn7803US103, complete genome	**KJ019069.1**	**NC_026926.1**	[4]	2013
				Synechococcus phage ACG-2014j isolate Syn7803US23, complete genome	KJ019089.1	-	[4]	2013
Syn	**S-CAM7**	1883368	2	Synechococcus phage S-CAM7 isolate 0910CC49, complete genome	**KU686212.1**	**NC_031927.1**	[6]	2016
				Synechococcus phage S-CAM7 isolate 0910SB42, complete genome	KU686213.1	-	[6]	2016
Syn	**S-SM2**	444860	1	Synechococcus phage S-SM2, complete genome	**GU071095.1**	**NC_015279.1**	[1]	2009
Syn	**syn9**	382359	1	Synechococcus phage syn9, complete genome	**DQ149023.2**	**NC_008296.2**	[10]	2005
Syn	**S-RSM4**	555387	1	Synechococcus phage S-RSM4 complete genome	**FM207411.1**	**NC_013085.1**	[11]	2008
Syn	**Syn19**	445684	1	Synechococcus phage Syn19, complete genome	**GU071106.1**	**NC_015286.1**	[1]	2009
Syn	**S-SM1**	444859	1	Synechococcus phage S-SM1, complete genome	**GU071094.1**	**NC_015282.1**	[1]	2009
Syn	**S-ShM2**	445683	1	Synechococcus phage S-ShM2, complete genome	**GU071096.1**	**NC_015281.1**	[1]	2009
Syn	**S-SSM5**	445685	1	Synechococcus phage S-SSM5, complete genome	**GU071097.1**	**NC_015289.1**	[1]	2009
Syn	**S-SSM7**	445686	1	Synechococcus phage S-SSM7, complete genome	**GU071098.1**	**NC_015287.1**	[1]	2009
Syn	**Syn33**	444878	1	Prochlorococcus phage Syn33, complete genome	**GU071108.1**	**NC_015285.1**	[1]	2009
Syn	**Syn1**	444861	1	Prochlorococcus phage Syn1, complete genome	**GU071105.1**	**NC_015288.1**	[1]	2009
Syn	**metaG-MbCM1**	1079999	1	Synechococcus phage metaG-MbCM1, complete genome	**JN371769.1**	**NC_019443.1**	-	2010
Syn	**S-CRM01**	1026955	1	Synechococcus phage S-CRM01, complete genome	**HQ615693.1**	**NC_015569.1**	[12]	2010
Syn	**S-SKS1**	754042	1	Synechococcus phage S-SKS1 genomic sequence	**HQ633071.1**	**NC_020851.1**	-	2010
Syn	**Syn30**	536474	1	Cyanophage Syn30 genomic sequence	**HQ634189.1**	**NC_021072.1**	-	2010
Syn	**S-IOM18**	754039	1	Synechococcus phage S-IOM18 genomic sequence	**HQ317383.1**	**NC_021536.1**	-	2010
Syn	**S-SSM4**	536466	1	Cyanophage S-SSM4 genomic sequence	**HQ316583.1**	**NC_020875.1**	-	2010
Syn	**Syn2**	536473	1	Cyanophage Syn2 genomic sequence	**HQ634190.1**	-	-	2010
Syn	**Syn10**	536472	1	Cyanophage Syn10 genomic sequence	**HQ634191.1**	-	-	2010
Syn	**KBS-M-1A**	889950	1	Cyanophage KBS-M-1A genomic sequence	**JF974293.1**	-	-	2010
Syn	**S-SSM6a**	682650	1	Cyanophage S-SSM6a genomic sequence	**HQ317391.1**	-	-	2010
Syn	**S-SSM6b**	682651	1	Cyanophage S-SSM6b genomic sequence	**HQ316603.1**	-	-	2010
Syn	**S-SSM2**	536464	1	Cyanophage S-SSM2 genomic sequence	**JF974292.1**	-	-	2010
Syn	**S-TIM5**	1137745	1	Cyanophage S-TIM5, complete genome	**JQ245707.1**	**NC_019516.1**	[13]	2011
Syn	**S-MbCM100**	1340812	1	Synechococcus phage S-MbCM100, complete genome	**KF156340.1**	**NC_023584.1**	[7]	2013
Syn	**ACG-2014h**	1340810	1	Synechococcus phage S-MbCM7, complete genome	**KF156338.1**	**NC_023587.1**	[7]	2013
Syn	**ACG-2014g**	1493512	1	Synechococcus phage ACG-2014g isolate Syn7803US105, complete genome	**KJ019071.1**	**NC_026924.1**	-	2013
Syn	**ACG-2014i**	1493513	1	Synechococcus phage ACG-2014i isolate Syn7803US120, complete genome	**KJ019082.1**	**NC_027132.1**	[4]	2013
Syn	**S-MbCM25**	1340811	1	Synechococcus phage S-MbCM25, complete genome	**KF156339.1**	-	[7]	2013
Syn	**S-WAM1**	1815521	1	Synechococcus phage S-WAM1 isolate 0810PA09, complete genome	**KU686210.1**	**NC_031944.1**	[6]	2016
Syn	**S-WAM2**	1815522	1	Synechococcus phage S-WAM2 isolate 0810PA29, complete genome	**KU686211.1**	**NC_031935.1**	[6]	2016
Syn	**S-RIM32**	1278479	1	Cyanophage S-RIM32 isolate RW_108_0702, complete genome	**KU594606.1**	**NC_031235.1**	[6]	2016
Syn	**S-RIM50**	687803	1	Cyanophage S-RIM50 isolate RW_29_0704, complete genome	**KU594605.1**	**NC_031242.1**	[6]	2016
Syn	**S-CBWM1**	2053653	1	Synechococcus phage S-CBWM1, complete genome	**MG450654.1**	-	[14]	2017

Syn	**Bellamy**	2023996	1	Synechococcus phage Bellamy, complete genome	**MF351863.1**	-	-	2017
Syn	**S-H35**	1983572	1	Synechococcus phage S-H35, complete genome	**KY945241.1**	-	-	2017
Syn	**S-B68**	2545437	1	Synechococcus phage S-B68, complete genome	**MK016664.1**	-	-	2018
Syn	**S-T4**	2268578	1	Synechococcus phage S-T4, complete genome	**MH412654.1**	-	-	2018
Syn	**S-B64**	2163901	1	Synechococcus phage S-B64, complete genome	**MH107246.1**	-	-	2018
Syn	**S-PRM1**	2100130	1	Synechococcus virus S-PRM1, complete genome	**MH629685.1**	-	-	2018
Syn	**S-E7**	2484639	1	Synechoccus phage S-E7, complete genome	**MH920640.1**	-	-	2018
Syn	**S-B43**	1340812	1	Synechococcus phage S-B43, complete genome	**MN018232.1**	-	-	2019
Syn	**B3**	2674978	1	Synechococcus phage B3, complete genome	**MN695334.1**	-	-	2019
Syn	**B23**	2674977	1	Synechococcus phage B23, complete genome	**MN695335.1**	-	-	2019
Syn	**S-RIM4**	2530169	1	Cyanophage S-RIM4 isolate RW_11_0999, complete genome	**MK493321.1**	-	-	2019
Syn	**S-B05**	2484637	1	Synechococcus phage S-B05, complete genome	**MK799832.1**	-	[15]	2019
Syn	**S-H34**	2718942	1	Synechococcus phage S-H34, complete genome	**MT162467.2**	-	This study	2020
Syn	**S-N03**	2718943	1	Synechococcus phage S-N03, complete genome	**MT162466.1**	-	This study	2020

References

[1] Sullivan, M.B.; Huang, K.H.; Ignacio-Espinoza, J.C.; Berlin, A.M.; Kelly, L.; Weigele, P.R.; DeFrancesco, A.S.; Kern, S.E.; Thompson, L.R.; Young, S.; Yandava, C.; Fu, R.; Krastins, B.; Chase, M.; Sarracino, D.; Osburne, M.S.; Henn, M.R.; Chisholm, S.W. Genomic analysis of oceanic cyanobacterial myoviruses compared with T4-like myoviruses from diverse hosts and environments. *Environmental Microbiology* **2010**, *12, 3035-3056*. https://doi.org/10.1111/j.1462-2920.2010.02280.x

[2] Fridman, S.; Flores-Uribe, J.; Larom, S. et al. A myovirus encoding both photosystem I and II proteins enhances cyclic electron flow in infected Prochlorococcus cells. *Nat Microbiol* **2017**, *2, 1350–1357*. https://doi.org/10.1038/s41564-017-0002-9

[3] Marston, M.F.; Martiny, J.B.H. Genomic diversification of marine cyanophages into stable ecotypes. *Environmental Microbiology* **2016**, *18(11), 4240-4253*. https://doi.org/10.1111/1462-2920.13556

[4] Gregory, A.C.; Solonenko, S.A.; Ignacio-Espinoza, J.C.; LaButti, K.; Copeland, A.; Sudek, S.; ... & Worden, A.Z. Genomic differentiation among wild cyanophages despite widespread horizontal genetransfer. *BMC Genomics* **2016**, *17(930)*. https://doi.org/10.1186/s12864-016-3286-x

[5] Marston, M.F.; Pierciey, F.J.; Shepard, A.; Gearin, G.; Qi, J.; Yandava, C.; ... & Martiny, J.B. Rapid diversification of coevolving marine *Synechococcus* and a virus. *Proceedings of the National Academy of Sciences of the United States of America* **2012**, *109(12), 4544-4549*. https://doi.org/10.1073/pnas.1120310109

[6] Crummett, L.T.; Puxty, R.J.; Weihe, C. The genomic content and context of auxiliary metabolic genes in marine cyanomyoviruses. *Virology* **2016**, *499, 219-229.* https://doi.org/10.1016/j.virol.2016.09.016

[7] Deng, L.; Ignacio-Espinoza, J.C.; Gregory, A.C.; Poulos, B.T.; Weitz, J.S.; Hugenholtz, P. & Sullivan, M.B. Viral tagging reveals discrete populations in *Synechococcus*

viral genome sequence space. *Nature* **2014**, *513(7517), 242–245*. https://doi.org/10.1038/nature13459
[8] Mann, N.H.; Clokie, M.R.J.; Millard, A.; Cook, A.; Wilson, W.H.; Wheatley, P.J.; Letarov, A.; Krisch, M.H. The Genome of S-PM2, a "Photosynthetic" T4-Type Bacteriophage That Infects Marine *Synechococcus* Strains. *Journal of Bacteriology* **2005**, *187(9), 3188-3200*. https://doi.org/10.1128/JB.187.9.3188-3200.2005
[9] Puxty, R.J.; Perez-Sepulveda.; Rihtman, B.; Evans, D.J.; Millard, A.D.; Scanlan, D.J. Spontaneous Deletion of an "ORFanage" Region Facilitates Host Adaptation in a "Photosynthetic" Cyanophage. *PLOS ONE* **2015**, *10(7), e0132642-*. https://doi.org/10.1371/journal.pone.0132642
[10] Weigele, P.R.; Pope, W.H.; Pedulla, M.L.; Houtz, J.M.; Smith, A.L.; Conway, J.F.; King, J.; Hatfull, G.F.; Lawrence, J.G.; Hendrix, R.W. Genomic and structural analysis of Syn9, a cyanophage infecting marine *Prochlorococcus* and *Synechococcus*. *Environmental Microbiology* **2007**, *9, 1675-1695*. https://doi.org/10.1111/j.1462-2920.2007.01285.x
[11] Millard, A.D.; Zwirglmaier, K.; Downey, M.J.; Mann, N.H.; Scanlan, D.J. Comparative genomics of marine cyanomyoviruses reveals the widespread occurrence of *Synechococcus* host genes localized to a hyperplastic region: implications for mechanisms of cyanophage evolution. *Environmental Microbiology* **2009**, *11(9), 2370-2387*. https://doi.org/10.1111/j.1462-2920.2009.01966.x
[12] Dreher, T.W.; Brown, N.; Bozarth, C.B.; Schwartz, A.D.; Riscoe, E.; Thrash, J.C.; Bennett, S.E.; Tzeng, S.C.; Maier, C.S. A freshwater cyanophage whose genome indicates close relationships to photosynthetic marine cyanomyophages. *Environmental Microbiology* **2011**, *13(7), 1858–1874*. https://doi.org/10.1111/j.14622920.2011.02502.x
[13] Sabehi, G.; Shaulov, L.; Silver, D.H.; Yanai, I.; Harel, A.; Lindell, D. A novel lineage of myoviruses infecting cyanobacteria is widespread in the oceans. *Proceedings of the National Academy of Sciences of the United States of America* **2012,** *109(6), 2037-2042.* https://doi.org/10.1073/pnas.1115467109
[14] Xu, Y.; Zhang, R.; Wang, N.; Cai, L.; Tong, Y.; Sun, Q.; Chen, F.; Jiao, N. Novel phage–host interactions and evolution as revealed by a cyanomyovirus isolated from an estuarine environment. *Environ Microbiol* **2018**, *20, 2974-2989.* https://doi.org/10.1111/1462-2920.14326
[15] Jiang, T.; Guo, C.; Wang, M.; Wang, M.; You, S.; Liu, Y.; ... & McMinn, A. Isolation and complete genome sequence of a novel cyanophage, S-B05, infecting an estuarine Synechococcus strain: insights into environmental adaptation. *Archives of Virology* **2020**, *165(6), 1397-1407.* https://doi.org/10.1007/s00705-020-04595-6
