# Supplementary material for: Genome Analysis of Two Novel Synechococcus Phages That Lack Common Auxiliary Metabolic Genes: Possible Reasons and Ecological Insights by Comparative Analysis of Cyanomyoviruses
Source: Viruses. 2020 Jul 25;12(8):800. doi: 10.3390/v12080800 (PMC7472177; doi:10.3390/v12080800)
Supplement: Supplementary file 1 [file viruses-12-00800-s001.zip › Supplementary Materials Table S3.pdf]

Supplementary Materials Table S3. Predicted ORFs in the S-N03 genome with homologues in the non-redundant database

ORF	strand	start	stop	Function	Match Phage	E-value	aa identity	Accession	Conserved domains accession
1	-	2708	852	baseplate wedge subunit	[Synechococcus phage S-B68]	0.00E+00	65%	QBP06140.1	PHA02553
2	-	3156	2746	T4-like baseplate wedge	[Synechococcus phage S-CRM01]	3.00E-45	52%	YP_004508434.1	pfam04965
3	-	3337	3185	hypothetical protein	no hits	1.00E-01			
4	-	3527	3330	hypothetical protein	[Synechococcus phage S-B68]	1.00E-30	75%	QBP06137.1	
5	-	4542	3583	tail fiber protein	[Synechococcus phage S-B28]	3.00E-07	36%	QBP05809.1	
6	-	5761	4568	putative tail fiber protein	[Prokaryotic dsDNA virus sp.]	4.00E-09	31%	QDP66378.1	pfam13884
7	-	6473	5793	hypothetical protein	[uncultured Mediterranean phage uvMED]	8.00E-06	30%	BAR33462.1	
8	-	7936	6512	tail fiber protein	[Synechococcus phage S-B28]	1.00E-16	33%	QBP05809.1	
9	-	9084	7975	tail fiber protein	[Synechococcus phage S-B28]	6.00E-22	30%	QBP05809.1	
10	-	9359	9102	hypothetical protein	[Prochlorococcus phage P-TIM68]	6.00E-08	34%	YP_009213543.1	
11	-	10100	9390	hypothetical protein	[uncultured Mediterranean phage uvMED]	4.00E-06	27%	BAR33462.1	
12	-	11361	10132	tail fiber protein	[Synechococcus phage S-B28]	5.00E-09	26%	QBP05809.1	
13	-	12436	11393	tail fiber protein	[Synechococcus phage S-B28]	3.00E-07	36%	QBP05809.1	
14	-	13109	12465	hypothetical protein	[uncultured Mediterranean phage uvMED]	9.00E-16	30%	BAR33462.1	
15	-	14781	13147	tail fiber protein	[Synechococcus phage S-B28]	7.00E-42	40%	QBP05809.1	
16	-	16752	14845	hypothetical protein	[Synechococcus phage S-B05]	3.00E-47	65%	QCW22942.1	PTZ00121
17	-	16987	16778	hypothetical protein	[Synechococcus phage S-B68]	1.00E-04	34%	QBP06132.1	
18	-	17506	17057	hypothetical protein	[Synechococcus phage S-B68]	4.00E-76	77%	QBP06130.1	
19	-	18022	17813	hypothetical protein					
20	-	18217	18023	hypothetical protein					
21	-	19159	18584	hypothetical protein	[Synechococcus phage S-B68]	5.00E-19	30%	QBP06128.1	
22	+	19207	19656	hypothetical protein		1.00E-03			pfam01541
23	-	19910	19677	hypothetical protein	[Synechococcus phage S-B68]	1.00E-19	64%	QBP06127.1	
24	-	20029	19907	hypothetical protein		5.50E+00			
25	-	20429	20196	hypothetical protein	[Synechococcus phage S-B68]	2.00E-08	38%	QBP06125.1	
26	-	20990	20889	hypothetical protein	no hits				

27	-	21114	20977	hypothetical protein	no hits			
28	-	21266	21111	hypothetical protein	no hits			
29	-	21493	21344	hypothetical protein	no hits	6.80E+00		
30	-	21690	21490	hypothetical protein	no hits			
31	-	22048	21809	hypothetical protein	[Synechococcus virus P60]	2.00E-19	49%	NP_570357.1
32	-	22236	22045	hypothetical protein	no hits			
33	-	22475	22239	hypothetical protein	no hits	3.40E+00		
34	-	22649	22482	hypothetical protein	[Synechococcus phage S-CAM1]	2.00E-13	60%	YP_007672954.1
35	-	22994	22815	hypothetical protein	no hits			
36	-	23333	23067	hypothetical protein	[Prochlorococcus phage P-HM2]	1.00E-08	38%	YP_004323429.1
37	-	23560	23372	hypothetical protein	[Synechococcus phage S-B68]	8.00E-13	48%	QBP06113.1
38	-	24199	23642	hypothetical protein	[Synechococcus phage S-B68]	4.00E-06	30%	QBP06110.1
39	-	24767	24282	hypothetical protein	no hits			
40	-	25331	24918	hypothetical protein	no hits	2.00E-03		
41	-	25474	25328	hypothetical protein	no hits			
42	-	25680	25474	hypothetical protein	no hits	4.50E+00		
43	-	26301	25951	hypothetical protein	[Acinetobacter phage ABPH49]	1.00E-04	31%	AXN57854.1
44	-	26569	26423	hypothetical protein	no hits			
45	-	26761	26573	hypothetical protein	no hits			
46	-	26993	26793	hypothetical protein	no hits			
47	-	27408	27070	hypothetical protein	[Synechococcus phage S-B68]	2.00E-15	42%	QBP06024.1
48	-	27557	27411	hypothetical protein	no hits			
49	-	27754	27560	hypothetical protein	no hits			
50	-	27912	27754	hypothetical protein	[Synechococcus phage S-B68]	3.00E-12	57%	QBP06116.1
51	-	28281	27994	hypothetical protein	[Synechococcus phage S-B68]	2.00E-11	37%	QBP06090.1
52	-	28384	28283	hypothetical protein	no hits			
53	-	29587	28457	hypothetical protein	no hits			
54	-	29787	29653	hypothetical protein	no hits	2.00E-03		
55	-	30283	29780	hypothetical protein	[Synechococcus phage S-B68]	3.00E-90	74%	QBP06064.1
56	-	30539	30303	hypothetical protein	no hits	5.00E+00		
57	-	30899	30774	hypothetical protein	no hits			
58	-	31012	30899	hypothetical protein	no hits	4.40E-01		

59	-	31218	31021	hypothetical protein	no hits				
60	-	31590	31294	hypothetical protein	no hits				
61	-	32465	31587	hypothetical protein	[Synechococcus phage S-B68]	2.00E-121	60%	QBP06092.1	
62	-	32722	32531	hypothetical protein	no hits				
63	-	32916	32719	hypothetical protein	no hits	3.00E-03			
64	-	33119	32913	hypothetical protein	no hits	8.90E+00			
65	-	33376	33122	hypothetical protein	no hits				
66	-	33648	33376	hypothetical protein	[Synechococcus phage S-B68]	4.00E-27	53%	QBP06089.1	
67	-	33886	33638	hypothetical protein	[Synechococcus phage S-CAM9]	1.00E-12	36%	YP_009322657.1	
68	-	34092	33883	hypothetical protein	no hits	2.60E-01			
69	-	34646	34092	hypothetical protein	[Synechococcus phage S-B68]	3.00E-26	41%	QBP06088.1	
70	-	34974	34714	hypothetical protein	[Synechococcus phage S-B68]	1.00E-08	43%	QBP06087.1	
71	-	35622	35041	2OG-Fe(II) oxygenase	[Prochlorococcus phage P-SSM7]	5.00E-20	36%	YP_004324864.1	pfam13640
72	-	36152	35622	hemagglutinin domain-containing protein	[Synechococcus phage S-B68]	2.00E-63	54%	QBP06086.1	
73	-	36435	36217	hypothetical protein	[Synechococcus phage S-B68]	5.00E-27	75%	QBP06085.1	
74	-	36952	36554	hypothetical protein	[Cyanophage S-RIM4]	7.00E-07	37%	QBQ74942.1	
75	-	37221	36955	hypothetical protein	no hits	9.90E+00			
76	-	37364	37224	hypothetical protein	no hits				
77	-	37694	37434	hypothetical protein	[Synechococcus phage S-B68]	5.00E-28	62%	QBP06082.1	
78	-	37941	37717	hypothetical protein	no hits	4.30E+00			
79	-	38135	38046	hypothetical protein	no hits	1.30E-01			
80	-	38370	38218	hypothetical protein	[Synechococcus phage S-B68]	5.00E-18	71%	QBP06074.1	
81	-	38782	38444	hypothetical protein	no hits				
82	-	39776	39012	subfamily RNA polymerase sigma-70 subunit	[Synechococcus phage ACG-2014f]	6.00E-38	35%	AIX32103.1	pfam04542
83	-	40052	39849	hypothetical protein	[Synechococcus phage S-B68]	9.00E-11	56%	QBP06071.1	
84	-	40284	40054	hypothetical protein	no hits				
85	-	40687	40340	hypothetical protein	[Synechococcus phage S-B68]	9.00E-10	38%	QBP06070.1	
86	-	41071	40763	hypothetical protein	no hits				
87	-	41474	41103	hypothetical protein	[Synechococcus phage S-CRM01]	8.00E-04	34%	YP_004508591.1	
88	-	41754	41521	hypothetical protein	[Synechococcus phage S-B68]	5.00E-25	66%	QBP06055.1	
89	-	41965	41747	hypothetical protein	no hits				

90	-	42267	41995	hypothetical protein	[Synechococcus phage S-B68]	3.00E-16	35%	QBP06057.1	
91	-	42461	42270	hypothetical protein	no hits				
92	-	42601	42458	hypothetical protein	[uncultured Mediterranean phage uvMED]	1.00E-10	51%	BAR33677.1	
93	-	42827	42606	hypothetical protein	[Synechococcus phage S-SSM7]	2.00E-19	61%	YP_004324343.1	
94	-	43063	42824	hypothetical protein	[Prokaryotic dsDNA virus sp.]	7.00E-09	48%	QDP62795.1	
95	-	43453	43211	hypothetical protein	[Synechococcus phage S-CAM7]	5.00E-23	55%	AOV62409.1	
96	-	43747	43508	hypothetical protein	[Synechococcus phage S-B68]	1.00E-23	67%	QBP06112.1	
97	-	43985	43821	hypothetical protein	no hits				
98	+	44144	44329	hypothetical protein	no hits				
99	+	44329	44640	hypothetical protein	[Synechococcus phage S-B68]	1.00E-26	55%	QBP06065.1	
100	+	44637	45449	Ser/Thr protein phosphatase family protein	[Synechococcus phage S-B68]	3.00E-86	51%	QBP06068.1	pfam00149
101	+	45433	45705	hypothetical protein	[Synechococcus phage S-B68]	3.00E-31	0%	QBP06069.1	
102	+	45738	45884	hypothetical protein	no hits				
103	+	45911	46942	hypothetical protein	no hits	1.50E+00			
104	+	47015	47419	hypothetical protein	no hits				
105	+	47416	47883	hypothetical protein	[Synechococcus phage S-B68]	5.00E-18	63%	QBP06052.1	pfam11753
106	+	47932	48597	sliding clamp DNA polymerase accessory protein	[Synechococcus phage S-B68]	2.00E-137	85%	QBP06051.1	PHA02545
107	+	48826	48981	hypothetical protein	no hits	6.70E+00			
108	+	48984	49103	hypothetical protein	no hits				
109	+	49166	50098	replication factor C small subunit / DNA polymerase clamp loader subunit	[Synechococcus phage S-B68]	1.00E-174	73%	QBP06046.1	PHA02544
110	+	50221	50769	homing endonuclease	[Synechococcus phage S-B43]	7.00E-09	49%	QDH50703.1	
111	+	50806	51174	clamp loader subunit	[Synechococcus phage S-CRM01]	1.00E-29	43%	YP_004508586.1	PHA02593
112	+	51190	51363	hypothetical protein	[Synechococcus phage S-B68]	7.00E-07	49%	QBP06044.1	
113	+	51367	51513	hypothetical protein	no hits				
114	+	51573	51971	endoribonuclease translational repressor of early genes	[Synechococcus phage S-CAM7]	1.00E-63	65%	YP_009323106.1	PHA02543
115	+	52061	52465	hsp20 small heat shock protein	[Synechococcus phage S-RSM4]	2.00E-42	54%	YP_003097310.1	COG0071
116	+	52531	52869	hypothetical protein	[Synechococcus phage S-B68]	3.00E-27	46%	QBP06040.1	
117	+	52838	53221	hypothetical protein	[Synechococcus phage S-CRM01]	1.00E-14	39%	YP_004508576.1	
118	+	53240	54706	DNA polymerase	[Synechococcus phage S-B68]	0.00E+00	80%	QBP06038.1	PHA02528

119	+	54703	54948	hypothetical protein	[Synechococcus phage ACG-2014f]	2.00E-09	46%	AIX20345.1	
120	+	54926	56026	DNA polymerase	[Synechococcus phage S-B68]	0.00E+00	83%	QBP06038.1	smart00486
121	+	56057	57061	RecA-like (recombination) protein	[Synechococcus phage S-B68]	0.00E+00	89%	QBP06036.1	COG0468
122	+	57063	57701	hypothetical protein	[Prochlorococcus phage P-TIM68]	2.00E-35	35%	YP_009213663.1	TIGR02466
123	+	57770	58135	hypothetical protein	[Synechococcus phage ACG-2014i]	4.00E-21	46%	YP_009140992.1	
124	+	58119	59510	DNA primase/helicase	[Synechococcus phage S-B68]	0.00E+00	80%	QBP06035.1	COG0305
125	+	59513	59923	pyrophosphatase（MazG	[Synechococcus phage S-B68]	3.00E-84	86%	QBP06034.1	cd11541
126	+	59923	60111	hypothetical protein	[Synechococcus phage S-B68]	4.00E-23	65%	QBP06028.1	
127	+	60185	60820	hypothetical protein	[Synechococcus phage S-CAM7]	2.00E-79	62%	AOV62347.1	TIGR00730
128	+	60817	61107	hypothetical protein	[Synechococcus phage S-CAM7]	3.00E-39	63%	AOV62358.1	
129	+	61132	61386	hypothetical protein	no hits				
130	+	61481	62104	plastoquinol terminal oxidase (PTOX)	[Prochlorococcus phage P-HM1]	3.00E-09	32%	YP_004322645.1	cd01053
131	+	62174	62794	NADPH-dependent oxidoreductase	[Harvfovirus sp.]	3.00E-09	29%	AYV80983.1	COG0431
132	+	62865	63188	hypothetical protein	no hits				
133	+	63185	63358	hypothetical protein	no hits				
134	+	63361	63585	hypothetical protein	[Synechococcus phage ACG-2014g]	2.00E-08	40%	YP_009133607.1	TIGR01575
135	+	63924	64370	hypothetical protein	[Synechococcus phage S-B68]	3.00E-34	44%	QBP06020.1	
136	+	64370	66430	serine/threonine kinase PknB	[Synechococcus phage S-B68]	2.00E-147	42%	QBP06019.1	cd05120
137	+	66448	66675	hypothetical protein	[Synechococcus phage S-B68]	5.00E-20	61%	QBP06018.1	
138	+	66675	69593	hypothetical protein	[Synechococcus phage ACG-2014f]	0.00E+00	63%	AIX27538.1	
139	+	69644	70537	DNA adenine methylase	[Synechococcus phage ACG-2014f]	3.00E-176	77%	AIX27539.1	COG0338
140	+	70534	70674	hypothetical protein	[Synechococcus phage ACG-2014f]	1.00E-06	50%	YP_009134382.1	
141	+	70696	71808	hypothetical protein	[Prochlorococcus phage P-TIM68]	3.00E-25	49%	YP_009213635.1	
142	+	71845	71970	hypothetical protein	[Synechococcus phage ACG-2014f]	9.40E-02	39%	AIX31845.1	
143	+	71963	72994	recombination-related endonuclease	[Synechococcus phage S-B68]	0.00E+00	76%	QBP06014.1	PHA02546
144	+	72991	73302	hypothetical protein	[Synechococcus phage S-B68]	6.00E-24	46%	QBP06013.1	pfam11360
145	+	73299	75020	recombination-related endonuclease	[Synechococcus phage S-B68]	0.00E+00	75%	QBP06012.1	PHA02562
146	-	75371	75048	PAAR protein	[Synechococcus phage S-B68]	2.00E-42	86%	QBP06010.1	pfam05488

147	-	75594	75361	hypothetical protein	[Synechococcus phage S-B68]	6.00E-10	48%	QBP06009.1	
148	-	76400	75600	hypothetical protein	[Synechococcus phage S-B68]	2.00E-20	31%	QBP06008.1	
149	-	77514	76462	hypothetical protein	[Synechococcus phage S-B68]	2.00E-130	60%	QBP06007.1	PHA02596
150	-	80192	77511	putative base plate hub subunit and tail lysozyme	[Synechococcus phage S-CRM01]	2.00E-138	43%	YP_004508492.1	PHA02596
151	-	81448	80189	hypothetical protein	[Synechococcus phage S-B68]	0.00E+00	66%	QBP06005.1	
152	-	83616	81448	hypothetical protein	[Synechococcus phage S-CRM01]	1.00E-12	39%	YP_004508494.1	cd00737
153	-	86417	83616	hypothetical protein	[Prochlorococcus phage P-TIM68]	4.00E-13	34.27%	YP_009213524.1	COG3179
154	-	86637	86449	base plate hub assembly catalyst	[Synechococcus phage S-CRM01]	6.00E-24	65%	YP_004508495.1	PHA02078
155	-	87350	86643	baseplate hub subunit	[Synechococcus phage S-CRM01]	6.00E-90	52%	YP_004508496.1	pfam12322
156	-	87851	87396	head completion protein	[Synechococcus phage S-B68]	3.00E-90	82%	QBP06000.1	PHA02552
157	+	87888	88511	hypothetical protein	[Synechococcus phage S-B68]	3.00E-72	54%	QBP05999.1	PHA02551
158	+	88511	88852	hypothetical protein	[Synechococcus phage S-B68]	6.00E-43	58%	QBP05998.1	PHA02577
159	+	88849	89709	baseplate tail tube cap	[Synechococcus phage S-CRM01]	3.00E-87	49%	YP_004508500.1	
160	+	89709	90350	baseplate wedge protein	[Synechococcus phage S-CRM01]	6.00E-63	46%	YP_004508501.1	PHA02578
161	-	90559	90386	hypothetical protein	no hits				
162	-	90789	90658	hypothetical protein	[Cyanophage S-RIM4]	9.70E-01	59%	QBQ75062.1	
163	-	91718	90789	single stranded DNA-binding protein	[Synechococcus phage S-B68]	3.00E-170	77%	QBP05995.1	PHA02550
164	-	92200	91796	hypothetical protein					
165	-	92808	92200	loader of DNA helicase	[Synechococcus phage S-CAM7]	3.00E-57	40%	YP_009323198.1	PHA02559
166	-	93079	92810	late promoter transcriptional accessory protein	[Synechococcus phage S-B68]	1.00E-31	62%	QBP05992.1	pfam16805
167	-	93758	93072	exonuclease A	[Synechococcus phage S-B68]	8.00E-99	63%	QBP05991.1	TIGR00372
168	-	94498	93758	phosphate starvation-inducible protein	[Synechococcus phage S-B68]	6.00E-144	77%	QBP05990.1	pfam02562
169	-	94749	94498	hypothetical protein	[Synechococcus phage S-CRM01]	6.00E-34	67%	YP_004508511.1	
170	-	94940	94749	hypothetical protein	[Synechococcus phage S-B68]	5.00E-21	63%	QBP05988.1	
171	-	95602	94934	thymidylate synthase	[Synechococcus phage S-B68]	2.00E-131	80%	QBP05987.1	pfam02511
172	-	95772	95599	hypothetical protein	[Cyanophage S-RIM4]	5.00E-05	51%	QBQ74938.1	
173	-	96339	95836	hypothetical protein	[Synechococcus phage S-B68]	5.00E-102	85%	QBP05984.1	
174	-	97258	96416	ribonuclease H	[Synechococcus phage S-B68]	8.00E-162	75%	QBP05983.1	PHA02567

175	-	97616	97341	hypothetical protein	[Synechococcus phage S-B68]	2.00E-23	57%	QBP05982.1	
176	-	97993	97757	glutaredoxin	[Synechococcus phage S-B68]	3.00E-27	64%	QBP05980.1	cd03029
177	-	98296	98105	hypothetical protein	[Synechococcus phage S-B68]	2.00E-24	63%	QBP05979.1	
178	-	98433	98293	hypothetical protein	no hits				
179	-	99016	98426	NusG antitermination factor	[Synechococcus phage S-CRM01]	8.00E-63	54%	YP_004508537.1	COG0250
180	-	99228	99016	hypothetical protein	[Synechococcus phage S-B68]	9.00E-21	57%	QBP05976.1	PHA02360
181	-	99585	99421	hypothetical protein	[Synechococcus phage S-B68]	4.00E-12	56%	QBP05972.1	
182	-	99999	99622	hypothetical protein	no hits				
183	-	100479	100186	hypothetical protein	[Synechococcus phage ACG-2014f]	2.00E-25	52%	AIX41699.1	
184	-	100687	100547	hypothetical protein	no hits				
185	-	101340	101185	hypothetical protein	[Synechococcus phage S-SM2]	2.00E-07	43%	YP_004322390.1	
186	-	101737	101354	putative endonuclease	[Prochlorococcus phage P-SSM2]	3.00E-42	55%	YP_214523.1	pfam14279
187	-	102078	101860	hypothetical protein	[Synechococcus phage S-B68]	1.00E-16	52%	QBP06195.1	
188	-	102227	102105	hypothetical protein	[Synechococcus phage S-B68]	1.00E-06	49%	QBP06163.1	
189	-	102373	102230	hypothetical protein	no hits				
190	-	102612	102370	hypothetical protein	no hits				
191	-	102891	102652	hypothetical protein	[Synechococcus phage S-B68]	3.00E-27	64%	QBP06194.1	PHA02325
192	-	103273	102881	hypothetical protein	[Synechococcus phage ACG-2014f]	9.00E-13	34%	AIX42712.1	
193	-	103956	103300	hypothetical protein	[Synechococcus phage S-B68]	8.00E-76	57%	QBP06192.1	cd00736
194	-	105333	104065	endolysin	[Synechococcus phage S-B68]	5.00E-169	58%	QBP06191.1	COG3179
195	-	105674	105330	hypothetical protein	[Synechococcus phage S-B68]	1.00E-59	75%	QBP06190.1	
196	-	105812	105708	hypothetical protein	no hits				
197	-	105928	105812	hypothetical protein	[Synechococcus phage S-SSM7]	1.00E-04	53%	YP_004324338.1	pfam14105
198	-	111076	106175	putative tail fiber-like protein	[Synechococcus virus S-PRM1]	4.00E-21	41%	AXN58441.1	
199	-	112555	111209	hypothetical protein	[Synechococcus phage S-B68]	2.00E-12	39%	QBP06153.1	
200	-	113679	112618	ribonucleotide reductase subunit B	[Synechococcus phage S-CRM01]	0.00E+00	71%	YP_004508549.1	COG0208
201	-	116000	113679	ribonucleotide-diphosphate reductase subunit A	[Synechococcus phage S-CRM01]	0.00E+00	69%	YP_004508552.1	PHA02572
202	-	116998	115997	DNA primase	[Synechococcus phage S-CRM01]	1.00E-141	60%	YP_004508553.1	PHA02540
203	-	117272	117045	structural protein	[Synechococcus phage S-B68]	5.00E-08	85%	QBP06184.1	

204	-	117643	117272	hypothetical protein	[Synechococcus phage S-B68]	3.00E-72	79%	QBP06183.1	PHA02335
205	-	118190	117666	hypothetical protein	[Synechococcus phage S-B68]	5.00E-40	56%	QBP06182.1	PTZ00357
206	-	118943	118230	hypothetical protein	[Synechococcus phage S-B68]	8.00E-146	84%	QBP06181.1	
207	-	120652	118940	hypothetical protein	[Synechococcus phage S-CAM7]	9.00E-11	46%	YP_009323180.1	
208	-	121557	120682	hypothetical protein	[Synechococcus phage S-B68]	5.00E-148	70%	QBP06179.1	cd02039
209	-	121696	121544	hypothetical protein	no hits				
210	-	122281	121793	RNA polymerase sigma factor for late transcription	[Synechococcus phage S-B68]	2.00E-99	85%	QBP06178.1	PHA02547
211	-	122830	122411	hypothetical protein	[Synechococcus phage S-B68]	3.00E-33	41%	QBP06177.1	pfam16243
212	-	124311	122827	RNA-DNA + DNA-DNA helicase	[Synechococcus phage S-B68]	0.00E+00	87%	QBP06176.1	cd17926
213	-	124485	124336	hypothetical protein	[Synechococcus phage ACG-2014h]	5.00E-04	46%	YP_009008194.1	
214	-	124712	124482	hypothetical protein	[Synechococcus phage S-B68]	3.00E-06	36%	QBP06174.1	
215	-	125137	124712	single-stranded DNA binding protein	[Cyanophage S-RIM12]	4.00E-33	42.96%	AOO15182.1	pfam11056
216	-	125834	125169	tail completion protein	[Synechococcus phage S-B68]	3.00E-107	68%	QBP06172.1	PHA02576
217	-	126323	125859	hypothetical protein	[Lactobacillus phage phiLdb]	4.00E-09	47%	YP_008770171.1	TIGR01453
218	-	127793	126405	major capsid protein	[Synechococcus phage S-B68]	0.00E+00	77%	QBP06171.1	PHA02541
219	-	128903	127842	prohead assembly (scaffolding) protein	[Synechococcus phage S-B68]	0.00E+00	86%	QBP06170.1	PHA02557
220	-	129632	128991	prohead core scaffolding protein and protease	[Synechococcus phage S-CRM01]	2.00E-112	75%	YP_004508474.1	PHA00911
221	-	129825	129616	hypothetical protein	[Synechococcus phage S-B68]	6.00E-32	79%	QBP06168.1	
222	-	131625	129862	portal vertex of the head	[Synechococcus phage S-B68]	0.00E+00	76%	QBP06167.1	PHA02531
223	-	132312	131713	tail tube protein	[Synechococcus phage S-B68]	8.00E-112	76%	QBP06166.1	PHA02551
224	-	134751	132343	tail sheath monomer	[Synechococcus phage S-B68]	0.00E+00	74%	QBP06165.1	pfam17482
225	-	135108	134884	hypothetical protein	no hits				
226	-	136823	135108	terminase large subunit	[Synechococcus phage S-B68]	0.00E+00	86%	QBP06164.1	PHA02533
227	-	138369	136867	hypothetical protein	[Synechococcus phage S-B68]	1.00E-116	40%	QBP06162.1	
228	-	139105	138413	hypothetical protein	[Synechococcus phage S-B68]	2.00E-30	41%	QBP06161.1	PTZ00121
229	-	139344	139108	hypothetical protein	no hits				
230	-	139799	139365	terminase small subunit	[Synechococcus phage S-B68]	2.00E-63	64%	QBP06159.1	PHA02585
231	-	140365	139796	hypothetical protein	[Synechococcus phage S-B68]	1.00E-35	41%	QBP06037.1	

232	-	141211	140381	tail sheath stabilizer	[Synechococcus phage S-CRM01]	5.00E-121	60%	YP_004508464.1	PHA02556
233	-	142269	141235	neck protein	[Synechococcus phage S-CRM01]	2.00E-120	51%	YP_004508463.1	PHA02555
234	-	143418	142276	neck protein	[Synechococcus phage S-CRM01]	3.00E-134	51%	YP_004508462.1	PHA02554
235	-	143560	143438	hypothetical protein	[uncultured Mediterranean phage uvMED]	2.00E-05	49%	BAR34689.1	
236	-	145142	143637	baseplate wedge tail fiber connector	[Synechococcus phage S-SSM5]	5.00E-22	51%	YP_004324698.1	
237	-	145582	145289	hypothetical protein	[Synechococcus phage S-CBP2]	5.00E-11	33%	YP_009103152.1	
238	-	145830	145585	hypothetical protein	[Synechococcus phage S-B28]	4.00E-18	49%	QBP05808.1	
239	-	147021	145879	hypothetical protein	[Synechococcus phage S-CRM01]	3.00E-13	31%	YP_004508451.1	
240	-	147597	147049	hypothetical protein	[uncultured Mediterranean phage uvMED]	2.00E-09	28%	BAQ93967.1	
241	-	150611	147597	hypothetical protein	[Synechococcus phage S-B68]	2.00E-157	46%	QBP06149.1	pfam13884
242	-	151060	150650	hypothetical protein	[Synechococcus phage S-B68]	1.00E-34	46%	QBP06147.1	
243	-	151350	151066	hypothetical protein	[Synechococcus phage S-B68]	3.00E-32	58%	QBP06146.1	
244	-	152701	151376	tail collar protein	[Synechococcus phage S-CAM8]	4.00E-45	36%	AET72703.1	pfam07484
245	-	152939	152703	hypothetical protein	[Synechococcus phage S-B68]	1.00E-26	68%	QBP06144.1	
246	-	160429	152999	structural protein	[Synechococcus phage S-CRM01]	0.00E+00	44%	YP_004508442.1	pfam16075
247	-	161954	160482	baseplate wedge subunit	[Synechococcus phage S-B68]	0.00E+00	55%	QBP06142.1	PHA02580
