# Supplementary material for: Genome Analysis of Two Novel Synechococcus Phages That Lack Common Auxiliary Metabolic Genes: Possible Reasons and Ecological Insights by Comparative Analysis of Cyanomyoviruses
Source: Viruses. 2020 Jul 25;12(8):800. doi: 10.3390/v12080800 (PMC7472177; doi:10.3390/v12080800)
Supplement: Supplementary file 1 [file viruses-12-00800-s001.zip › Supplementary Materials Table S4.pdf]

Supplementary Materials Table S4. Predicted ORFs in the S-H34 genome with homologues in the non-redundant database

ORF	strand	start	stop	Function	Match Phage	E-value	aa identity	Accession	Conserved domains accession
1	-	461	3	hypothetical protein	[Synechococcus phage S-B68]	8.00E-92	92.16%	QBP06154.1	
2	-	1948	467	hypothetical protein	[Synechococcus phage S-B68]	0.00E+00	85.56%	QBP06153.1	
3	-	3093	2032	ribonucleotide reductase subunit B	[Synechococcus phage S-CRM01]	0.00E+00	71.09%	YP_004508549.1	PRK09101
4	-	5395	3095	ribonucleotide-diphosphate reductase subunit A	[Synechococcus phage S-CRM01]	0.00E+00	69.66%	YP_004508552.1	PHA02572
5	-	6393	5392	DNA primase	[Synechococcus phage S-CRM01]	1.00E-144	61.52%	YP_004508553.1	PHA02540
6	-	7219	6440	hypothetical protein	[Synechococcus phage S-SKS1]	7.00E-67	72.97%	YP_007674514.1	
7	-	7590	7219	hypothetical protein	[Synechococcus phage S-B68]	6.00E-72	81.30%	QBP06183.1	PHA02335
8	-	8136	7609	hypothetical protein	[Synechococcus phage S-B68]	6.00E-17	45.28%	QBP06182.1	
9	-	8886	8173	hypothetical protein	[Synechococcus phage S-B68]	4.00E-143	83.05%	QBP06181.1	PLN02853
10	-	10562	8883	hypothetical protein	[Synechococcus phage S-CAM7]	2.00E-10	46.30%	YP_009323180.1	
11	-	11464	10592	cytidylyltransferase	[Synechococcus phage S-H68]	3.00E-57	39.47%	QBP05852.1	TIGR00125
12	-	11603	11451	hypothetical protein	no hit				
13	-	12152	11667	RNA polymerase sigma factor for late transcription	[Synechococcus phage S-B68]	3.00E-94	83.97%	QBP06178.1	PHA02547
14	-	12698	12282	methylamine utilization protein	[Synechococcus phage S-SM2]	1.00E-19	36.76%	YP_004322281.1	pfam16243
15	-	14182	12695	RNA-DNA + DNA-DNA helicase	[Synechococcus phage S-B68]	0.00E+00	88.08%	QBP06176.1	PHA02558
16	-	14362	14207	hypothetical protein	[Synechococcus phage ACG-2014h]	1.00E-07	52.27%	YP_009008194.1	
17	-	14574	14359	hypothetical protein	no hit				
18	-	14999	14574	single-stranded DNA binding protein UvsY	[Cyanophage S-RIM12]	8.00E-33	41.55%	AOO15182.1	pfam11056
19	-	15696	15031	tail completion and sheath stabilizer	[Synechococcus phage S-CRM01]	6.00E-91	57.01%	YP_004508478.1	PHA02576
20	-	16209	15718	hypothetical protein	no hit				
21	-	17686	16313	major capsid protein	[Synechococcus phage S-B68]	0.00E+00	78.85%	QBP06171.1	PHA02541
22	-	18795	17734	prohead core scaffold protein	[Synechococcus phage S-CRM01]	8.00E-141	59.39%	YP_004508476.1	PHA02557
23	-	19523	18882	prohead core scaffolding protein and protease	[Synechococcus phage S-CRM01]	2.00E-112	75.12%	YP_004508474.1	PHA00911
24	-	19716	19507	hypothetical protein	[Synechococcus phage S-B68]	3.00E-31	77.46%	QBP06168.1	
25	-	21519	19753	portal vertex head protein	[Synechococcus phage S-CRM01]	0.00E+00	68.47%	YP_004508471.1	PHA02531
26	-	22206	21607	tail tube protein	[Synechococcus phage S-B68]	1.00E-112	75.38%	QBP06166.1	PHA02551

27	-	24663	22237	tail sheath monomer	[Synechococcus phage S-B68]	0.00E+00	74.32%	QBP06165.1	PHA02539
28	-	26512	24806	terminase large subunit	[Synechococcus phage S-B68]	0.00E+00	88.89%	QBP06164.1	PHA02533
29	-	28950	26536	tail fiber protein	[Synechococcus phage S-B68]	2.00E-61	39.02%	QBP06148.1	PHA02584
30	-	29442	29011	putative integration and excision endonuclease VII	[Synechococcus phage S-H68]	2.00E-14	31.85%	QBP05926.1	pfam02945
31	-	30919	29498	hypothetical protein	[Synechococcus phage S-B68]	5.00E-130	45.07%	QBP06162.1	
32	-	31604	30963	hypothetical protein	[Synechococcus phage S-B68]	6.00E-29	42.04%	QBP06161.1	PTZ00121
33	-	32058	31621	terminase small subunit	[Synechococcus phage S-B68]	6.00E-64	64.10%	QBP06159.1	PHA02585
34	-	32891	32061	tail sheath stabilizer	[Synechococcus phage S-CRM01]	2.00E-126	62.45%	YP_004508464.1	PHA02556
35	-	33957	32920	neck protein	[Synechococcus phage S-CRM01]	3.00E-115	49.29%	YP_004508463.1	PHA02555
36	-	34629	33976	homing endonuclease	[Synechococcus phage S-B43]	9.00E-19	29.05%	QDH50703.1	cd00085
37	-	35492	34650	neck protein	[Synechococcus phage S-CRM01]	4.00E-60	49.49%	YP_004508462.1	PHA02554
38	-	35651	35511	hypothetical protein	[Synechococcus phage S-B68]	1.00E-06	65.22%	QBP06155.1	
39	-	37215	35710	baseplate wedge tail fiber connector	[Synechococcus phage S-SSM5]	4.00E-24	51.35%	YP_004324698.1	
40	-	37669	37337	hypothetical protein	[Synechococcus phage S-CRM01]	3.00E-10	58.54%	YP_004508467.1	
41	-	37977	37684	hypothetical protein	[Synechococcus phage S-CBP2]	1.00E-13	36.08%	YP_009103152.1	
42	-	38225	37980	hypothetical protein	[Synechococcus phage S-B28]	4.00E-20	53.09%	QBP05808.1	
43	-	39440	38274	hypothetical protein	[Synechococcus phage S-CRM01]	4.00E-10	29.21%	YP_004508451.1	
44	-	40016	39468	hypothetical protein	[uncultured Mediterranean phage uvMED]	3.00E-09	28.22%	BAQ93967.1	
45	-	42994	40016	hypothetical protein	[Synechococcus phage S-B68]	4.00E-155	45.50%	QBP06149.1	pfam13884
46	-	43443	43033	hypothetical protein	[Synechococcus phage S-B68]	4.00E-39	48.53%	QBP06147.1	
47	-	43733	43449	hypothetical protein	[Synechococcus phage S-B68]	2.00E-30	56.00%	QBP06146.1	
48	-	45081	43759	tail collar domain containing protein	[Synechococcus phage S-CRM01]	4.00E-91	38.46%	YP_004508444.1	pfam07484
49	-	45319	45083	hypothetical protein	[Synechococcus phage S-B68]	3.00E-21	59.21%	QBP06144.1	
50	-	52816	45383	structural protein	[Synechococcus phage S-CRM01]	0.00E+00	44.37%	YP_004508442.1	pfam16075
51	-	54342	52870	baseplate wedge subunit	[Synechococcus phage S-B68]	0.00E+00	55.17%	QBP06142.1	PHA02580
52	-	60215	54387	structural protein	[Cyanophage S-RIM44]	4.00E-98	28.32%	AOO12976.1	pfam14240
53	-	62068	60212	baseplate wedge subunit	[Synechococcus phage S-B68]	0.00E+00	64.78%	QBP06140.1	PHA02553
54	-	62515	62105	base plate wedge subunit	[Synechococcus phage ACG-2014f]	3.00E-30	48.44%	AIX42332.1	PHA00415
55	-	62696	62544	hypothetical protein	no hit				
56	-	62886	62689	hypothetical protein	[Synechococcus phage S-B68]	2.00E-31	75.38%	QBP06137.1	
57	-	63961	62942	hypothetical protein	no hit				

58	-	65000	63987	tail fiber	[Synechococcus phage ACG-2014f]	5.00E-12	33.47%	AIX42737.1	pfam13884
59	-	65694	65032	hypothetical protein	[Synechococcus phage S-RIP2]	7.00E-05	33.04%	YP_007673155.1	
60	-	67358	65733	putative tail fiber protein	[Synechococcus phage S-B43]	1.00E-41	34.75%	QDH50628.1	
61	-	68527	67397	putative tail fiber protein	[Synechococcus phage S-B43]	3.00E-28	31.93%	QDH50628.1	
62	-	68802	68545	hypothetical protein	[Prochlorococcus phage P-TIM68]	7.00E-08	34.43%	YP_009213543.1	
63	-	69522	68833	hypothetical protein	[uncultured Mediterranean phage uvMED]	1.00E-13	31.21%	BAQ93188.1	
64	-	70852	69554	tail fiber protein	[Synechococcus phage S-B28]	2.00E-36	37.41%	QBP05809.1	
65	-	71945	70884	tail fiber protein	[Synechococcus phage S-B28]	2.00E-15	28.08%	QBP05809.1	
66	-	72699	71974	hypothetical protein	[uncultured Mediterranean phage uvMED]	6.00E-16	32.44%	BAR33462.1	
67	-	74278	72737	hypothetical protein	[Prochlorococcus phage P-TIM68]	1.00E-30	33.94%	YP_009213536.1	
68	-	76122	74344	hypothetical protein	[Synechococcus phage S-IOM18]	4.00E-46	93.33%	YP_008126475.1	PTZ00121
69	-	76335	76147	hypothetical protein	no hit				
70	-	76853	76407	hypothetical protein	[Synechococcus phage S-B68]	2.00E-64	66.2%	QBP06130.1	cd16339
71	-	76984	76850	hypothetical protein	no hit				
72	-	77369	77160	hypothetical protein	no hit				
73	-	77560	77366	hypothetical protein	no hit				
74	-	78492	77914	hypothetical protein	[Synechococcus phage S-B68]	6.00E-22	32.54%	QBP06128.1	
75	-	79001	78489	hypothetical protein	[Synechococcus phage ACG-2014f]	7.00E-06	35.58%	AIX44871.1	
76	-	79337	79014	hypothetical protein	[Synechococcus phage S-B68]	3.00E-17	67.27%	QBP06127.1	
77	-	79573	79334	hypothetical protein	[Synechococcus phage S-B68]	9.00E-12	39.74%	QBP06125.1	
78	+	80200	80322	hypothetical protein	no hit				
79	-	80521	80348	hypothetical protein	[Synechococcus phage S-CAM7]	4.00E-20	62.26%	YP_009322998.1	
80	-	80675	80526	hypothetical protein	no hit				
81	-	80797	80672	hypothetical protein	no hit				
82	-	80994	80794	hypothetical protein	no hit				
83	-	81322	81113	hypothetical protein	[Synechococcus phage S-CAM1]	1.00E-12	57.14%	YP_007672954.1	
84	-	81667	81488	hypothetical protein	no hit				
85	-	82006	81740	hypothetical protein	[Prochlorococcus phage P-HM2]	6.00E-08	36.14%	YP_004323429.1	
86	-	82251	82042	hypothetical protein	[Synechococcus phage S-B68]	2.00E-09	39.34%	QBP06113.1	
87	-	82909	82334	hypothetical protein	no hit				
88	-	83474	83061	hypothetical protein	no hit				

89	-	83616	83482	hypothetical protein	no hit			
90	-	83822	83616	hypothetical protein	no hit			
91	-	84011	83871	hypothetical protein	no hit			
92	-	84413	84063	hypothetical protein	no hit			
93	-	84910	84494	hypothetical protein	no hit			
94	-	85176	85033	hypothetical protein	no hit			
95	-	85368	85180	hypothetical protein	no hit			
96	-	85598	85398	hypothetical protein	no hit			
97	-	85988	85668	hypothetical protein	[Synechococcus phage S-B68]	4.00E-20	39.81%	QBP06024.1
98	-	86134	85988	hypothetical protein	no hit			
99	-	86346	86167	hypothetical protein	[Synechococcus phage S-B68]	2.00E-12	59.09%	QBP06116.1
100	-	86715	86428	hypothetical protein	[Synechococcus phage S-B68]	3.00E-09	34.78%	QBP06090.1
101	-	86849	86712	hypothetical protein	no hit			
102	-	87345	86842	hypothetical protein	[Synechococcus phage S-B68]	2.00E-91	76.19%	QBP06064.1
103	-	87613	87365	hypothetical protein	no hit			
104	-	87970	87845	hypothetical protein	no hit			
105	-	88083	87970	hypothetical protein	no hit			
106	-	88280	88092	hypothetical protein	no hit			
107	-	88583	88344	hypothetical protein	no hit			
108	-	89458	88580	hypothetical protein	[Synechococcus phage S-B68]	2.00E-121	59.93%	QBP06092.1
109	-	90417	89542	hypothetical protein	no hit			
110	-	90665	90483	hypothetical protein	no hit			
111	-	90859	90662	hypothetical protein	[Synechococcus phage S-B68]	9.00E-05	47.83%	QBP06093.1
112	-	91062	90856	hypothetical protein	no hit			
113	-	91313	91059	hypothetical protein	no hit			
114	-	91579	91310	hypothetical protein	[Synechococcus phage S-B68]	6.00E-29	55.95%	QBP06089.1
115	-	91817	91569	hypothetical protein	[Synechococcus virus S-PRM1]	5.00E-14	39.06%	AXN58427.1
116	-	92023	91814	hypothetical protein	no hit			
117	-	92580	92023	hypothetical protein	[Synechococcus phage S-B68]	1.00E-24	38.85%	QBP06088.1
118	-	92954	92649	hypothetical protein	no hit			
119	-	93215	93021	hypothetical protein	[Synechococcus phage S-B68]	3.00E-05	45.76%	QBP06087.1
120	-	93745	93215	hemagglutinin domain-containing protein	[Synechococcus phage S-B68]	7.00E-64	55.49%	QBP06086.1

121	-	94029	93808	hypothetical protein	[Synechococcus phage S-B68]	2.00E-21	61.64%	QBP06085.1	
122	-	94493	94095	hypothetical protein	no hit				
123	-	94763	94497	hypothetical protein	no hit				
124	-	94903	94763	hypothetical protein	no hit				
125	-	95163	94906	hypothetical protein	[Synechococcus phage S-B68]	3.00E-30	68.83%	QBP06082.1	
126	-	95409	95185	hypothetical protein	no hit				
127	-	95608	95513	hypothetical protein	no hit				
128	-	95843	95691	hypothetical protein	[Synechococcus phage S-B68]	1.00E-12	62.75%	QBP06074.1	
129	-	96010	95918	hypothetical protein	no hit				
130	-	96912	96148	RNA polymerase sigma factor RpoD	[Cyanophage KBS-S-2A]	3.00E-14	29.50%	YP_007674984.1	TIGR02997
131	-	97210	97013	hypothetical protein	[Synechococcus phage S-B68]	5.00E-10	62.50%	QBP06071.1	
132	-	97425	97213	hypothetical protein	no hit				
133	-	97850	97503	hypothetical protein	[Synechococcus phage S-B68]	6.00E-07	35.63%	QBP06070.1	
134	-	98611	98240	hypothetical protein	no hit				
135	-	98891	98658	hypothetical protein	[Synechococcus phage S-B68]	3.00E-25	72.58%	QBP06055.1	
136	-	99181	98927	hypothetical protein	[Synechococcus phage S-B68]	4.00E-15	41.46%	QBP06057.1	
137	-	99351	99181	hypothetical protein	no hit				
138	-	99500	99354	hypothetical protein	[uncultured Mediterranean phage uvMED]	3.00E-06	47.22%	BAQ93270.1	
139	-	99727	99497	hypothetical protein	[Synechococcus phage S-B68]	1.00E-20	61.19%	QBP06058.1	
140	-	99963	99724	hypothetical protein	no hit				
141	-	100279	100037	hypothetical protein	[Synechococcus phage Bellamy]	2.00E-25	61.43%	ASR76234.1	
142	-	100612	100334	hypothetical protein	[Synechococcus phage S-B68]	1.00E-23	65.22%	QBP06112.1	
143	-	100853	100647	hypothetical protein	no hit				
144	-	101087	100923	hypothetical protein	no hit				
145	+	101227	101379	hypothetical protein	no hit				
146	+	101379	101690	hypothetical protein	[Synechococcus phage S-B68]	8.00E-30	57.14%	QBP06065.1	
147	+	101687	102478	Ser/Thr protein phosphatase family protein	[Synechococcus phage S-B68]	7.00E-86	50.6%	QBP06068.1	cd07404
148	+	102475	102588	hypothetical protein	no hit				
149	+	102612	102734	hypothetical protein	[Synechococcus phage S-B68]	1.00E-12	72.97%	QBP06069.1	
150	+	102767	102910	hypothetical protein	no hit				
151	+	102985	103983	hypothetical protein	no hit				

152	+	104053	104490	hypothetical protein	[Synechococcus phage S-B68]	1.00E-18	56.25%	QBP06052.1	pfam11753
153	+	104540	105205	sliding clamp DNA polymerase accessory protein	[Synechococcus phage S-B68]	5.00E-138	85.97%	QBP06051.1	PHA02545
154	+	105450	106547	DNA polymerase clamp loader subunit	[Synechococcus phage S-CAM3]	2.00E-91	45.71%	YP_009321424.1	PHA02544
155	+	106544	106690	hypothetical protein	no hit				
156	+	106668	107036	DNA polymerase clamp loader subunit	[Cyanophage S-RIM12]	8.00E-22	41.32%	AOO15845.1	PHA02593
157	+	107067	107246	hypothetical protein	[Synechococcus phage S-B68]	7.00E-07	51.11%	QBP06044.1	
158	+	107251	107403	hypothetical protein	no hit				
159	+	107459	107863	translation repressor	[Synechococcus phage ACG-2014g]	1.00E-59	65.65%	YP_009133701.1	PHA02543
160	+	107936	108451	hypothetical protein	[Synechococcus phage ACG-2014f]	4.00E-07	33.33%	AIX30722.1	pfam07463
161	+	108528	108932	Hsp20 heat shock protein	[Synechococcus phage ACG-2014e]	3.00E-37	50.77%	YP_009134640.1	pfam00011
162	+	108991	109341	hypothetical protein	[Synechococcus phage S-B68]	2.00E-28	46.00%	QBP06040.1	
163	+	109310	109684	hypothetical protein	[Synechococcus phage S-CRM01]	1.00E-14	42.53%	YP_004508576.1	
164	+	109696	111162	DNA polymerase	[Synechococcus phage S-B68]	0.00E+00	80.91%	QBP06038.1	PHA02528
165	+	111159	111404	hypothetical protein	[Synechococcus phage ACG-2014f]	1.00E-09	46.43%	AIX41243.1	
166	+	111382	112482	DNA polymerase	[Synechococcus phage S-B68]	0.00E+00	84.40%	QBP06038.1	PHA02528
167	+	112513	113517	recombination protein	[Synechococcus phage S-CAM9]	0.00E+00	80.78%	YP_009322578.1	COG0468
168	+	113587	114978	DNA primase/helicase	[Synechococcus phage S-B68]	0.00E+00	80.35%	QBP06035.1	PHA02542
169	+	114981	115391	pyrophosphatase	[Synechococcus phage S-B68]	7.00E-91	93.38%	QBP06034.1	pfam03819
170	+	115391	115564	hypothetical protein	[Synechococcus phage S-B05]	6.00E-13	52.73%	QCW22932.1	
171	+	115561	115749	hypothetical protein	[Cyanophage [Syn30]	8.00E-26	76.67%	YP_007877812.1	
172	+	115753	116388	hypothetical protein	[Synechococcus phage S-CAM7]	3.00E-84	67.04%	YP_009323093.1	COG1611
173	+	116385	116675	hypothetical protein	[Synechococcus phage ACG-2014f]	5.00E-39	62.50%	AIX31789.1	
174	+	116797	116955	hypothetical protein	no hit				
175	+	117026	117349	hypothetical protein	no hit				
176	+	117346	117519	hypothetical protein	no hit				
177	+	117522	117746	hypothetical protein	[Synechococcus phage ACG-2014g]	2.00E-08	40.26%	YP_009133607.1	
178	+	117746	117931	hypothetical protein	[Synechococcus phage S-B68]	3.00E-05	33.33%	QBP06021.1	
179	+	117950	118408	hypothetical protein	[Synechococcus phage S-B68]	3.00E-34	45.10%	QBP06020.1	
180	+	118408	120432	serine/threonine kinase PknB	[Synechococcus phage S-B68]	4.00E-155	42.61%	QBP06019.1	cd05120
181	+	120447	120686	hypothetical protein	[Synechococcus phage S-B68]	2.00E-18	55.70%	QBP06018.1	
182	+	120686	123619	hypothetical protein	[Synechococcus phage ACG-2014f]	0.00E+00	62.41%	AIX27538.1	

183	+	123670	124563	DNA adenine methylase	[Synechococcus phage ACG-2014f]	4.00E-175	76.95%	AIX27539.1	COG0338
184	+	124560	124700	hypothetical protein	[Synechococcus phage ACG-2014f]	4.00E-06	47.22%	YP_009134382.1	
185	+	124722	125669	hypothetical protein	[Prochlorococcus phage P-TIM68]	2.00E-27	51.18%	YP_009213635.1	
186	+	125706	125831	hypothetical protein	no hit				
187	+	125824	126855	recombination-related endonuclease	[Synechococcus phage S-B68]	0.00E+00	76.38%	QBP06014.1	PHA02546
188	+	126852	127163	hypothetical protein	[Synechococcus phage S-B68]	5.00E-25	44.68%	QBP06013.1	pfam11360
189	+	127160	128881	recombination-related endonuclease	[Synechococcus phage S-B68]	0.00E+00	75.22%	QBP06012.1	PHA02562
190	-	129199	128909	PAAR protein	[Synechococcus phage S-B68]	1.00E-42	87.34%	QBP06010.1	
191	-	129419	129183	hypothetical protein	[Synechococcus phage S-B68]	1.00E-26	67.53%	QBP06009.1	
192	-	130291	129416	hypothetical protein	[Synechococcus phage S-B68]	3.00E-24	33.11%	QBP06008.1	
193	-	131345	130293	hypothetical protein	[Synechococcus phage S-B68]	4.00E-131	60.24%	QBP06007.1	PHA02596
194	-	134047	131342	baseplate hub subunit and tail lysozyme	[Synechococcus phage S-CRM01]	1.00E-141	45.70%	YP_004508492.1	PHA02596
195	-	135303	134044	hypothetical protein	[Synechococcus phage S-B68]	0.00E+00	65.07%	QBP06005.1	
196	-	137510	135303	hypothetical protein	[Synechococcus phage S-CRM01]	3.00E-11	69.57%	YP_004508494.1	cd00737
197	-	140320	137510	hypothetical protein	[Prochlorococcus phage P-TIM68]	9.00E-15	36.03%	YP_009213524.1	COG3179
198	-	140539	140351	base plate hub assembly catalyst	[Synechococcus phage S-CRM01]	5.00E-24	62.07%	YP_004508495.1	PHA02078
199	-	141252	140545	baseplate assembly chaperone	[Synechococcus phage S-B68]	1.00E-124	73.62%	QBP06001.1	pfam12322
200	-	141754	141299	head completion protein	[Synechococcus phage S-B68]	2.00E-88	79.05%	QBP06000.1	PHA02552
201	+	141818	142411	hypothetical protein	[Synechococcus phage S-B68]	1.00E-70	53.81%	QBP05999.1	
202	+	142411	142749	hypothetical protein	[Synechococcus phage S-B68]	5.00E-42	61.06%	QBP05998.1	PHA02577
203	+	142746	143606	baseplate tail tube cap	[Synechococcus phage S-CRM01]	1.00E-85	47.24%	YP_004508500.1	
204	+	143611	144252	baseplate wedge protein	[Synechococcus phage S-CRM01]	4.00E-64	46.33%	YP_004508501.1	PHA02578
205	-	144460	144287	hypothetical protein	no hit				
206	-	144697	144566	hypothetical protein	no hit				
207	-	145626	144697	single stranded DNA-binding protein	[Synechococcus phage S-B68]	8.00E-172	78.59%	QBP05995.1	PHA02550
208	-	146108	145704	hypothetical protein	[Synechococcus phage S-B68]	1.00E-04	27.93%	QBP05994.1	
209	-	146716	146108	loader of gp41 DNA helicase	[Synechococcus phage S-CRM01]	9.00E-85	55.72%	YP_004508507.1	PHA02559
210	-	146987	146718	late promoter transcriptional accessory protein	[Synechococcus phage S-B68]	2.00E-31	61.80%	QBP05992.1	pfam16805
211	-	147666	146980	exonuclease A	[Synechococcus phage S-B68]	2.00E-100	63.72%	QBP05991.1	TIGR00372
212	-	148406	147666	phosphate starvation-inducible protein	[Synechococcus phage S-B68]	6.00E-144	77.46%	QBP05990.1	pfam02562
213	-	148657	148406	hypothetical protein	[Synechococcus phage S-CRM01]	1.00E-34	66.27%	YP_004508511.1	

214	-	148848	148657	hypothetical protein	[Synechococcus phage S-B68]	3.00E-22	66.67%	QBP05988.1	
215	-	149495	148842	thymidylate synthase	[Synechococcus phage S-B68]	2.00E-133	82.16%	QBP05987.1	PRK00847
216	-	149731	149492	hypothetical protein	[Cyanophage S-RIM4]	2.00E-04	48.57%	QBQ74938.1	
217	-	150231	149728	hypothetical protein	[Synechococcus phage S-B68]	3.00E-101	81.93%	QBP05984.1	
218	-	151076	150234	ribonuclease H	[Synechococcus phage S-B68]	1.00E-161	74.19%	QBP05983.1	PHA02567
219	-	151437	151159	hypothetical protein	[Synechococcus phage S-B68]	3.00E-23	56.00%	QBP05982.1	
220	-	151591	151442	hypothetical protein	no hit				
221	-	151823	151578	glutaredoxin	[Synechococcus phage S-B68]	3.00E-30	62.50%	QBP05980.1	pfam00462
222	-	152123	151932	hypothetical protein	[Synechococcus phage S-B68]	7.00E-25	65.08%	QBP05979.1	
223	-	152260	152120	hypothetical protein	no hit				
224	-	152843	152253	NusG antitermination factor	[Synechococcus phage S-CRM01]	5.00E-62	53.65%	YP_004508537.1	PRK05609
225	-	153109	152843	hypothetical protein	[Synechococcus phage S-B68]	8.00E-21	57.58%	QBP05976.1	PHA02360
226	-	153465	153301	hypothetical protein	[Synechococcus phage S-B68]	6.00E-12	59.62%	QBP05972.1	
227	-	153714	153502	hypothetical protein	[Synechococcus phage S-SKS1]	8.00E-13	40.58%	YP_007674557.1	
228	-	154073	153744	hypothetical protein	[Synechococcus phage S-H35]	2.00E-76	99.08%	QIN97099.1	
229	-	154456	154178	hypothetical protein	[Synechococcus phage ACG-2014f]	4.00E-29	56.38%	AIX27408.1	
230	-	154696	154526	hypothetical protein	no hit				
231	-	155411	155214	hypothetical protein	[Synechococcus phage ACG-2014f]	2.00E-20	67.92%	AIX21569.1	
232	+	155442	156164	hypothetical protein	[Synechococcus phage S-SKS1]	5.00E-31	34.39%	YP_007674511.1	
233	-	156550	156167	putative endonuclease	[Prochlorococcus phage P-SSM2]	4.00E-42	52.89%	YP_214523.1	pfam14279
234	-	156890	156666	hypothetical protein	[Synechococcus phage S-B68]	1.00E-16	45.33%	QBP06195.1	
235	-	157040	156918	hypothetical protein	[Synechococcus phage S-B68]	2.00E-05	40.00%	QBP06163.1	
236	-	157186	157043	hypothetical protein	no hit				
237	-	157347	157183	hypothetical protein	no hit				
238	-	158018	157587	hypothetical protein	[Synechococcus phage S-B68]	8.00E-41	51.37%	QBP06160.1	
239	-	158277	158044	hypothetical protein	[Synechococcus phage S-B68]	3.00E-37	77.63%	QBP06194.1	PHA02325
240	-	158659	158267	hypothetical protein	[Synechococcus phage ACG-2014f]	1.00E-14	34.43%	AIX21536.1	
241	-	159341	158685	hypothetical protein	[Synechococcus phage S-B68]	3.00E-73	54.42%	QBP06192.1	cd00736
242	-	160719	159451	endolysin	[Synechococcus phage S-B68]	7.00E-169	57.58%	QBP06191.1	COG3179
243	-	161060	160716	hypothetical protein	[Synechococcus phage S-B68]	7.00E-69	85.09%	QBP06190.1	
244	-	161196	161095	hypothetical protein	no hit				
245	-	161308	161204	hypothetical protein	no hit				

246	-	161439	161308	hypothetical protein	[Synechococcus phage S-WAM1]	8.00E-09	58.14%	YP_009325169.1	pfam14105
