# Supplementary figures and images for: Genome Analysis of Two Novel Synechococcus Phages That Lack Common Auxiliary Metabolic Genes: Possible Reasons and Ecological Insights by Comparative Analysis of Cyanomyoviruses
Source: Viruses. 2020 Jul 25;12(8):800. doi: 10.3390/v12080800 (PMC7472177; doi:10.3390/v12080800)

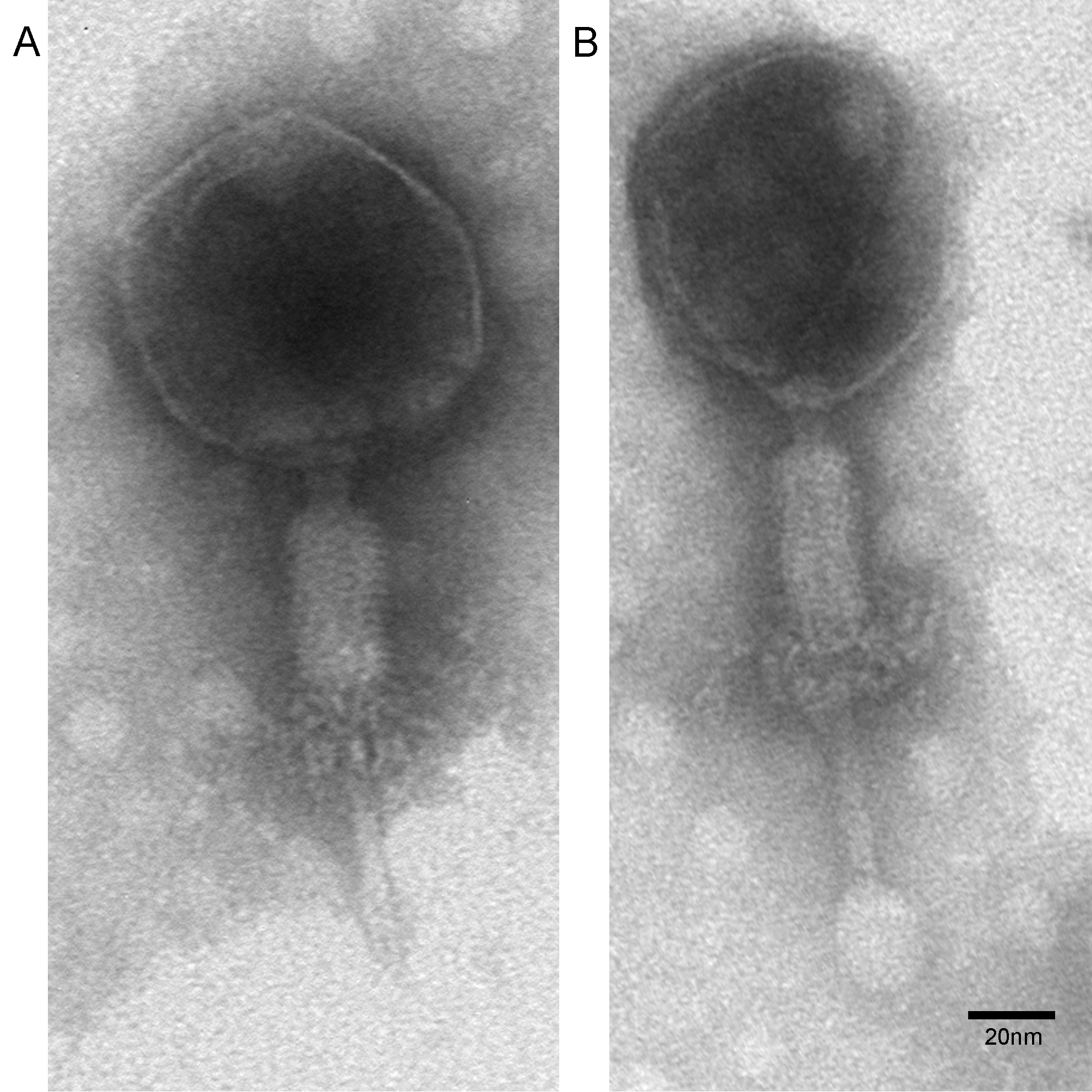

Supplement: Supplementary file 1 [file viruses-12-00800-s001.zip › Supplementary Materials Figure S1.tif]
